# Mitochondrial phosphate transporter and methyltransferase genes contribute to Fusarium head blight Type II disease resistance and grain development in wheat

**DOI:** 10.1371/journal.pone.0258726

**Published:** 2021-10-14

**Authors:** Keshav B. Malla, Ganesh Thapa, Fiona M. Doohan

**Affiliations:** UCD Earth Institute, UCD Institute of Food and Health and UCD School of Biology and Environmental Sciences, UCD Science Centre East, University College Dublin, Belfield, Dublin, Ireland; Institute of Genetics and Developmental Biology Chinese Academy of Sciences, CHINA

## Abstract

Fusarium head blight (FHB) is an economically important disease of wheat that results in yield loss and grain contaminated with fungal mycotoxins that are harmful to human and animal health. Herein we characterised two wheat genes involved in the FHB response in wheat: a wheat mitochondrial phosphate transporter (*TaMPT*) and a methyltransferase (*TaSAM*). Wheat has three sub-genomes (A, B, and D) and gene expression studies demonstrated that *TaMPT* and *TaSAM* homoeologs were differentially expressed in response to FHB infection and the mycotoxigenic *Fusarium* virulence factor deoxynivalenol (DON) in FHB resistant wheat cv. CM82036 and susceptible cv. Remus. Virus-induced gene silencing (VIGS) of either *TaMPT* or *TaSAM* enhanced the susceptibility of cv. CM82036 to FHB disease, reducing disease spread (Type II disease resistance). VIGS of *TaMPT* and *TaSAM* significantly reduced grain number and grain weight. This indicates *TaSAM* and *TaMPT* genes also contribute to grain development in wheat and adds to the increasing body of evidence linking FHB resistance genes to grain development. Hence, *Fusarium* responsive genes *TaSAM* and *TaMPT* warrant further study to determine their potential to enhance both disease resistance and grain development in wheat.

## Introduction

Fusarium head blight (FHB) is an economically important disease of wheat caused by *Fusarium* fungi. It reduces yield and contaminates grain with mycotoxins harmful to human and animal health, most commonly deoxynivalenol (DON) [1]. DON is also a virulence factor, facilitating the spread of *Fusarium* within wheat heads [2,3]. Many components of FHB resistance have been described, the most common being resistance to initial infection (Type I resistance) and resistance to disease spread (Type II resistance) and studies have shown that resistance to the deleterious effects of DON is a component of type II resistance in some wheat genotypes (reviewed in Gunupuru *et al*. [3]). Several studies have shown that specific processes and pathways are activated in wheat in response to DON [4–7]. UDP-glycosyltransferases (UGTs) gene involved in DON detoxification pathway have been shown to convert DON to less toxic DON-3-O-glucoside and overexpression of wheat *UGT* (*TaUGT3*) and barley *UGT* (*HvUGT13248)* increased DON tolerance in transgenic plants [4,8]. Genes involved in the classic detoxification pathway (drug transporters and cytochrome P450) have been shown to contribute to DON resistance in wheat, as well as the evolutionary divergent orphan gene *TaFROG* and the wheat sucrose non-fermenting-1 (SNF1)-related protein kinase 1 catalytic subunit α (SnRK1α) [9–12]. The diversity of pathways activated in response to DON in wheat [13] and barley [14] reaffirm the important role of this toxin in facilitating disease development.

Wheat genes encoding a mitochondrial phosphate transporter (*TaMPT*) and S-adenosyl methionine (SAM)-dependent methyltransferase (*TaSAM*) were identified as being responsive to DON based on a microarray analysis and were differentially expressed in cv. CM82036 x cv. Remus double haploid lines segregating for the FHB resistance QTL *Fhb1* [13]. But the role of these genes and their associated pathways in the wheat response to DON or to FHB is unknown. MPTs belong to the phosphate transporter 3 (PHT3) gene family. They are located in the inner membrane of mitochondria and are responsible for transporting inorganic phosphate (Pi) into the mitochondrial matrix, wherein the Pi is utilised for the oxidative phosphorylation of ADP to ATP [15–17]. MPT genes have been identified and characterised in many plant species, but studies on wheat MPT genes are very limited [18]. There is one report of the down-regulation of a MPT in wheat heads (resistant to FHB) in response to *F*. *graminearum* [19]. Other pathosystems also provide evidence for the involvement of MPTs in wheat disease responses. Yu et al. [20] identified two wheat *MPT* genes responsive to wheat stripe rust (*Puccinia striiformis*). Using microarray analysis, Xin et al. [21] showed that a *MPT* gene was differentially expressed in wheat in response to the causal agent of powdery mildew disease, *Blumeria graminis* f. sp. *tritici*, with higher expression in a resistant wheat line than in a susceptible line. Recently, genome-wide association studies (GWAS) and fine-mapping studies identified a *MPT* gene that co-segregated with the *Pch1* locus that confers resistance to eyespot disease caused by *Pseudocercosporella herpotrichoides* [22].

SAM-dependent methyltransferases enzymes catalyse the transfer of methyl groups from SAM to a large variety of acceptor substrates, ranging from small metabolites to bio-macromolecules [23]. These enzymes contain a cofactor (SAM) binding site and a substrate binding site and share little sequence identity [24]. Several studies have reported the responsive of wheat methyltransferase genes to *F*. *graminearum*. Gunnaiah et al. [25] demonstrated that the phenylpropanoid pathway genes encoding caffeic acid-O-methyltransferase, caffeoyl-CoA-O-methyltransferase, and flavonoid-O-methyltransferase were up-regulated in resistant wheat near-isogenic lines containing the FHB resistance QTL *Fhb1*. Schweiger et al. [26] fine-mapped and sequenced a 1Mb contig containing the *Fhb1* region from the FHB resistant cv. CM82036 and identified 28 candidate genes including a methyltransferase domain containing protein. However, the methyltransferase gene is unlikely an exclusive determinant of *Fhb1* resistance, since a deletion mutation in a histidine-rich calcium binding protein has been shown to confer *Fhb1* resistance [27]. Cho et al. [28] showed that a methyltransferase gene was differentially expressed in both the FHB resistant cultivar (cv.) Dahongmil and the susceptible cv. Urimil after inoculation with *F*. *graminearum*. Long et al. [29] found that a SAM-methyltransferase domain-encoding gene was one of eight candidates whose expression correlated with the FHB resistance QTL on chromosome 2D. Recently, AlTaweel et al. [30] highlighted a methyltransferase gene that was up-regulated in the presence of *F*. *graminearum* infection in FHB resistance cv. Sumai 3 and susceptible cv. Caledonia.

Herein, we characterised the mycotoxin-responsive *TaSAM* and the *TaMPT* genes first identified by Brennan et al. [13]. Homoeologous genes were identified on all wheat subgenomes and gene expression studies were conducted to determine their responsiveness to DON and DON-producing *F*. *graminearum* in the FHB resistant cv. CM82036 and susceptible wheat cv. Remus. Using VIGS, we determined the contribution of both *TaSAM* and the *TaMPT* genes to FHB disease resistance in wheat. Furthermore, the VIGS experiment assessed the contribution of *TaSAM* and *TaMPT* genes to grain development in wheat.

## Materials and methods

### Plant material, growth condition and fungal treatments

*Triticum aestivum* (wheat) cultivars (cvs.) CM82036 and Remus (obtained from Hermann Buerstmayr, BOKU), Chinese Spring and its’ derivative nullisomic-tetrasomic wheat lines (obtained from Germplasm Resources unit, JIC) were used in this study. ‘CM82036’ (derived from a ‘Sumai 3’/’Thornbird-S’ cross) is resistant to FHB and DON, and carries alleles for FHB resistance at two QTL, *Fhb1* (syn. *Qfhs*.*ndsu-3BS*) and syn *Qfhs*.*ifa-5A*. ‘Remus’ (derived from Sappo/Mex//Famos) is a German spring wheat cultivar and is susceptible to FHB [31,32]. Wheat seeds were germinated in darkness for 72 h at 24°C in 90 mm petri dishes containing moist Whatman No. 1 filter paper (Whatman, UK) and germinated seedlings were transferred to 3 litre pots containing John Innes compost No. 2 (Westland Horticulture, Dungannon, UK). Wheat studies were carried under contained glasshouse conditions with a day/night temperature of 25/18 °C and a light/dark regime of 16/8 h. The DON-producing *Fusarium graminearum* wild type strain GZ3639 [33] was cultured on potato dextrose agar (PDA) (Difco, UK) plates and incubated at 25°C for 5 days. Fungal spores were produced in mung bean broth [34], harvested, washed and adjusted to 10^6^ conidia ml^-1^ in 0.02% Tween-20, as previously described [35].

### Adult plant DON and FHB time course experiment

An adult plant experiment was conducted to analyse the temporal response of *TaMPT* and *TaSAM* genes homoeologs to both DON and FHB disease in the wheat cvs. CM82036 and Remus. At mid-anthesis (growth stage (GS) 65) [36], two central spikelets of heads from secondary tillers (and of similar size/number of spikelets) were inoculated with 20μl (40μl per head) of either deoxynivalenol (DON) (Santa Cruz, Texas, USA) (5mg ml^-1^ in 0.02% Tween-20) or 10^6^ conidia of *F*. *graminearum* strain GZ3639 [37] or 0.02% Tween-20 (mock treatment). After treatment, the heads were covered with a plastic bag for 48 hours to maintain high humidity. Treated spikelets were harvested at either 0, 12, 24, 48, 72, or 96 hours post-inoculation (hpi), flash-frozen in liquid nitrogen (N_2_) and stored at -70°C prior to RNA extraction. The experiment comprised three replicate trials (each conducted independently at different times), each including eight heads from four individual plants (two heads per plant) per treatment combination (therefore, across the independent trials, there was a total of 12 plants/24 heads per treatment combination). See S1 Table for experimental design. For gene expression studies, RNA was extracted from one pooled sample per treatment per trial (representing a pool of 8 heads from 4 individual plants per treatment per trial).

### DNA, RNA extraction and cDNA synthesis

DNA was extracted from wheat leaves using the HP plant DNA mini kit (OMEGA) following the manufacturer’s instructions. RNA was extracted from wheat heads as previously described [38] and was DNase-treated using the TURBO DNA-*free*TM kit (Ambion Inc., USA). The quality, yield and integrity of the RNA was analysed using both the ND-1000 spectrophotometer (NanoDrop, Thermo Fisher Scientific, USA) and electrophoresis. Reverse transcription of total RNA and the quality check of synthesized cDNA for DNA contamination was conducted as previously described [13].

### Cloning of *TaSAM-D* and *TaMPT-A* genes and promoters

Homoeolog-specific primers for the *TaSAM* from chromosome 2D (hereafter referred to as *TaSAM-D*) and *TaMPT* from chromosome 5A (hereafter referred to as *TaMPT-A*) genes and promoters were designed based on the cv. Chinese spring wheat reference sequence (IWGSC RefSeq V1.1) using genome-specific primers (GSPs), a web-based platform for designing genome-specific primers in polyploids [39]. For *TaMPT-A* promoter and gene cloning, primers were designed to amplify around 1500 bp upstream of the start codon and another set to amplify the coding region of the gene homoeologs (S2 Table). *TaSAM-D* primers was designed to amplify both the promoter and gene together as one product (S2 Table). Due to high sequence similarities between the 2A and 2B homoeologs of *TaSAM* gene it was not possible to design homoeolog-specific primers for these two genes. Homoeolog-specific primers for *TaSAM-D* gene and promoter were used to amplify targets from DNA of wheat cvs. CM82036 and Remus. PCR reactions contained 100 ng genomic DNA, 1.25 U of Takara Ex Taq^™^, 1X Ex Taq buffer (Mg^2+^ plus) and 2.5 mM of each dNTP in 50 μl reaction. Reaction conditions were as follows: 94°C for 5 min, 35 cycles of 94°C for 30 s, 55°C for 30 s, 68°C for 2 minutes and a final extension step at 72°C for 10 min and conducted in a ProFlex PCR System (Applied Biosystems by Life Technologies, USA). The amplified PCR products were cloned into the pCR^®^-XL-TOPO^®^ vector using the TOPO^®^ XL cloning kit (Invitrogen, UK) and sequenced using M13 forward and reverse primers. Results were validated for at least two independent PCR amplicons per target sequence. Sequences were aligned to IWGSC wheat genome database (https://wheat-urgi.versailles.inra.fr/Seq-Repository/BLAST) via BLASTn analysis.

### Sequence and phylogenetic analysis

*TaMPT-A* and *TaSAM-D* genes cloned from cvs. CM82036 and Remus were used to identify their homoeologs in wheat cv. Chinese spring via BLASTn analysis against Ensembl Genomes (http://plants.ensembl.org) and the wheat genome (IWGSC Refseq V1.1). Multiple sequence alignments of *TaMPT-A* and *TaSAM-D* genes and their homoeologs was constructed using MultAlin (http://multalin.toulouse.inra.fr/). *TaMPT-A* and *TaSAM-D* sequences from cvs. CM82036 and Remus were used to extract homologous sequences from other *Poaceae* via BLASTp against the EnsemblPlants database (http://plants.ensembl.org) [40]. Phylogenetic relationships of *TaMPT* and *TaSAM* homologs were deduced using the Neighbour-joining method with bootstrapping (1000 replicates) using the MEGA 7.0 program [41]. The sub-cellular localisation of the *TaSAM and TaMPT* genes was predicted using Multiloc2 [42]. For domain analysis, the amino acid sequence of *TaMPT and TaSAM* were used for BLASTp against the EnsemblPlants database and were further scanned using the TMHMMv 2.0 and the Interpro protein database [43].

### Virus-induced gene silencing (VIGS) constructs

The barley stripe mosaic virus (BSMV)-derived VIGS vectors used in this study consisted of the wild type BSMV ND18 α, β and γ tripartite genome [44,45]. For transient gene silencing, two independent, non-overlapping fragments were amplified for each gene: 194 and 177 bp for *TaSAM* and 171 and 145 bp for *TaMPT* (see S2 Table for PCR primers). The fragments were amplified from cv. CM82036 cDNA. Gene silencing fragments were selected to target all the homoeologs of either *TaMPT* (chromosome 5A, 5B and 5D genes) or *TaSAM* (chromosome 2A, 2B and 2D genes) (S3 Fig). The homology and gene silencing specificity of fragments for *TaMPT* homoeologs and *TaSAM* homoeologs was assessed via BLASTn analysis against the wheat genome and qRT-PCR using homoeolog-specific primers (S8 Table). For cloning the VIGS fragments, PCR reactions were performed with 30ng plasmid DNA, 1μM each of forward and reverse fragment-specific primers (S2 Table) in a 10μl reaction containing 0.5U Taq DNA polymerase and 1x PCR buffer (Invitrogen, UK), 1.5mM MgCl2, and 125μM of each dNTP. PCR reactions were conducted in a ProFlex PCR System (Applied Biosystems by Life Technologies, USA) and the PCR program consisted of an initial denaturation step at 94°C for 2 min, 35 cycles of 94°C for 30 s and 60°C for 30 s and a final extension step at 72°C for 5 min. The amplified silencing fragments were cloned into the pGEM-T vector (pGEM-T Easy cloning kit; Promega, UK). The pGEM-T vectors carrying the silencing fragments were digested with *NotI* and *PacI* sites (New England Biolabs, MA, USA), thereby generating *NotI* and *PacI* ends in DNA fragment. Inserts was purified by gel extraction and then the fragment was subsequently ligated to *NotI*- digested γ RNA vector pSL038-1 [45]. Plasmids containing the silencing fragments were sequenced by Macrogen (korea) using the vector-specific pGamma-F/R primers (S2 Table). A BSMV γ RNA vector carrying 185 bp fragment of barley phytoene desaturase (PDS) gene served as positive control [45]. The plasmids BSMV α and γ genome as well as γ RNA constructs with silencing fragments for *PDS*, *TaSAM* and *TaMPT* were linearized with Mlu1 restriction enzyme whereas BSMV β was linearized using the Spe1 enzyme. Linearized plasmids were converted into capped *in vitro* transcripts using mMessage Machine^™^ T7 in-vitro transcription kit (Ambion, Inc., Austin, TX, USA), following the manufacturer’s protocol. Once cloned into BSMV, these constructs were respectively named BSMV:SAM1 and BSMV:SAM2 for *TaSAM* and BSMV:MPT1 and BSMV:MPT2 for *TaMPT*.

### Virus-induced gene silencing of *TaMPT* and *TaSAM* genes

FHB resistant wheat cv. CM82036 plants were used for the VIGS experiment. The experiment consisted of three randomly designed independent trials (conducted at different times); each trial included 20 heads per treatment combination (10 plants, two heads per plant) (i.e. across the three independent trials there was a total of 30 plants/60 heads per treatment combination). See S1 Table for experimental design. Plants were grown as described above. At growth stage 47 [36] just before the emergence of the first wheat head, the flag leaves of secondary tillers (of similar size) were rub-inoculated with VIGS buffer FES or buffer containing a 1:1:1 mixture of the *in vitro* transcripts of BSMV α, β and γ RNA (BSMV:00) or derivatives γ RNA that contained plant fragments (BSMV:PDS, BSMV:SAM1, BSMV:SAM2, BSMV:MPT1, or BSMV:MPT2) [45]. At mid-anthesis (growth stage 65) [36], the two central spikelets of heads on VIGS-treated tillers were treated with either 10^6^ conidia of *F*. *graminearum* strain GZ3639 or 0.02% Tween-20 (mock treatment). Treated heads were covered with plastic bags for 2 days to maintain high humidity. The third spikelet above the treated spikelet was harvested 24h post-treatment, flash frozen in liquid N_2_ and stored at -70°C prior to RNA extraction (for gene expression studies). RNA was extracted from individual spikelets and equivalent amounts of RNA for the four treated heads per pot were bulked to give a total of five bulk RNA samples per treatment combination per trial (i.e. a total of 15 bulked RNA samples per treatment combination across all three trials). For all treated heads, the number of diseased (discoloured and necrotic) spikelets (including treated spikelets) was assessed at 14 and 21 days post-inoculation (dpi). At growth stage (GS 90), the treated heads were harvested and the average dry grain weight and number of seeds per head was determined.

### Quantitative Reverse Transcriptase PCR analysis

Quantitative real-time PCR (qRT-PCR) analyses were conducted using the Stratagene Mx3000TM Real Time PCR (Stratagene, Germany). Homoeolog-specific PCR primers used in this study (S2 Table) were designed using genome-specific primer (GSP) [46]. The specificity of the primers targeting the *TaMPT* and *TaSAM* homoeologs was checked via PCR of DNA extracts from nullisomic-tetrasomic lines of cv. Chinese Spring (obtained from Germplasm Resources Unit, JIC, Norwich http://www.jic.ac.uk/germplasm/). An off-target *TaMPT* gene variant located on chromosome 2 was also analysed to confirm VIGS specificity (see S2 Table for primers; this gene was chosen because it was next closest homolog and matches 20bp sequence identity with construct BSMV:MPT2). The efficiency of the qRT-PCR primers was checked via qPCR of a dilution series of samples. Each reaction contained 1.25 μl of a 1:5 (v/v) dilution of cDNA (1000–1.6 ng/μl), 0.2 μM of each primer and 1X SYBR^®^ Premix Ex Taq^™^ (Tli RNase H plus, RR420A, Takara) in a total reaction volume of 12.5 μl. PCR conditions were: 1 cycle of 1 min at 95°C; 40 cycles of 5 s at 95°C and 20 s at 60°C; and a final cycle of 1 min at 95°C, 30 s at 55°C and 30 s at 95°C for the dissociation curve. The threshold cycle (CT) values obtained were used to calculate the standard curve. The specificity of PCR amplification was confirmed by the presence of a single peak in melting temperature curve analysis of amplified fragments. Housekeeping genes used for wheat gene expression studies were *α-tubulin* (GenBank No. U76558.1) [47], Yellow-leaf specific gene 8 (YLS8, TraesCS1D02G332500) and protein phosphatase 2A subunit A3 *(TaPP2AA3*, TraesCS5B02G165200). These genes were verified not to be differentially expressed in the experiments or in publicly available RNA-seq studies for FHB experiments (results not shown). CT values obtained by real-time RT-PCR were used to calculate the relative gene expression using the formula 2^^-(CT target gene − CT housekeeping gene)^ as described previously [48]. For validation of virus-induced gene silencing, the same qRT-PCR conditions and homoeolog-specific primers were used, (the primers did not overlap with the VIGS construct sequences).

### Statistical analysis

All data were analysed using MINITAB 16 (Inc, 2010) (Minitab Ltd., Coventry, UK). Non-normally distributed data sets were transformed to fit a normal distribution using the Johnson transformation (Ryan & Joiner, 1976) and the statistical significance of difference was analysed using one-way analysis of variance incorporating Tukey’s test (*P* = 0.05). The data which could not be transformed using the Johnson transformation (Ryan & Joiner, 1976) was analysed using the non-parametric Mann-Whitney test in MINITAB.

## Results

### Analysis of *TaMPT* and *TaSAM* sequences and phylogeny

**Fig 1:**
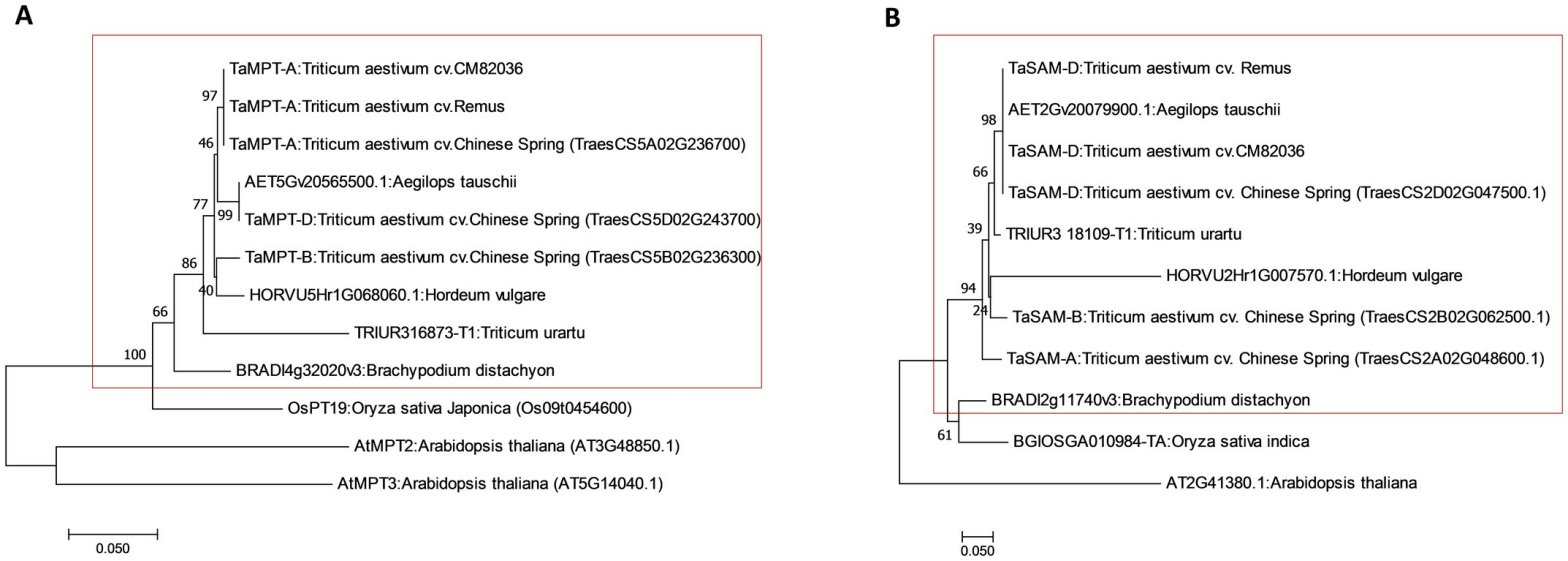
Phylogenetic analysis of *TaMPT* and *TaSAM* homologs across the plant kingdom. The deduced amino acid sequences of wheat cv. Chinese spring A) *TaMPT* genes, the 5A homoeologs from cvs. CM82036 and Remus, and the closest *Poaceae MPT* sequences and B) *TaSAM* genes, the 2D homoeologs from cvs. CM82036 and Remus, and the closest *Poaceae SAM* sequences obtained from Ensembl Plants were used for phylogenetic analysis. Phylogenetic analysis was constructed using Molecular Evolutionary Genetics Analysis Version 7 software (MEGA7) [41] as described previously [9]. Poisson correction method was used for computing the evolutionary distances and are in the units of the number of amino acid substitutions per site [49]. The consensus tree was inferred from 1000 bootstrap replicates. The branch lengths are in the same units as evolutionary distances used to infer the phylogenetic tree. Wheat and other *Pooideae* proteins are within the red box.

Previous studies within our laboratory identified novel *TaMPT* and *TaSAM* genes that were responsive to the toxigenic *Fusarium* mycotoxin DON (Walter et al., 2008). Based on homology, we deduced that the genes of interest were on chromosomes 5A and 2D, respectively, with homoeologs on the other wheat sub-genomes. The *TaMPT* gene on wheat chromosome 5A and *TaSAM* on 2D were cloned and sequenced from wheat cvs. CM82036 and Remus; sequences were then compared with homoeologs from the sequenced genome of cv. Chinese spring (IWGSC Ref seq v1.1). The *TaMPT-A* DNA sequence from cvs. CM82036 and Remus showed 100% identity with a sequence from cv. Chinese spring (CS) (S3 and S4 Tables). Two wheat homoeologs of *TaMPT-A* were identified by BLASTn analysis and are located on cv. Chinese spring chromosomes 5B and 5D, and are hereafter respectively referred to as *TaMPT-B* and *TaMPT-D*. Both homoeologs shared a high homology (> 98% nucleotide and amino acid identity) with the *TaMPT-A* (98.1 and 98.2% respectively; S3 and S4 Tables). *In silico* analysis of protein sequences predicted that all *TaMPT* homoeologs contain the mitochondrial carrier domain (IPR018108, S1 Fig and S5 Table). Multiloc2 predicted that *TaMPT* homoeologs have a high probability of localising to the mitochondria (S5 Table). The DNA sequence of *TaSAM-D* cloned from cvs. CM82036 and Remus showed 100% identity with a sequence on chromosome 2D of wheat cv. Chinese spring (S6 Table). Two homoeologs of *TaSAM-D* was located on cv. Chinese spring chromosomes 2A and 2B, and are hereafter referred to as *TaSAM-A* and *TaSAM-B*. Both share > 96% nucleotide and amino acid homology with *TaSAM-D* (S6 and S7 Tables). *In silico* sequence analysis predicted that all *TaSAM* homoeologs contain S-adenosyl-L-methionine-dependent methyltransferase and methyltransferase type 11 domains (IPR013216, S2 Fig and S8 Table). Multiloc2 predicted that *TaSAM* homoeologs have a high probability of localising to the cytoplasmic region (S8 Table). Phylogenetic analysis showed that wheat *TaMPT-A* and *TaSAM-D* proteins represent conserved gene families and these variants cluster with proteins from *Poaceae* plants (Fig 1A and 1B).

### *TaMPT* and *TaSAM* homoeologs are up-regulated in wheat heads in response to both DON and FHB disease

Gene expression studies analysed the response of *TaMPT* and *TaSAM* gene homoeologs to both DON and FHB in two wheat cultivars: one that is FHB and DON-resistant (cv CM82036), and one that is susceptible to both the toxin and the pathogen (cv. Remus). Transcripts of all homoeologous genes of *TaMPT* (5A, 5B, 5D) and *TaSAM* (2A, 2B, and 2D) were detected in heads of both cultivars.

**Fig 2:**
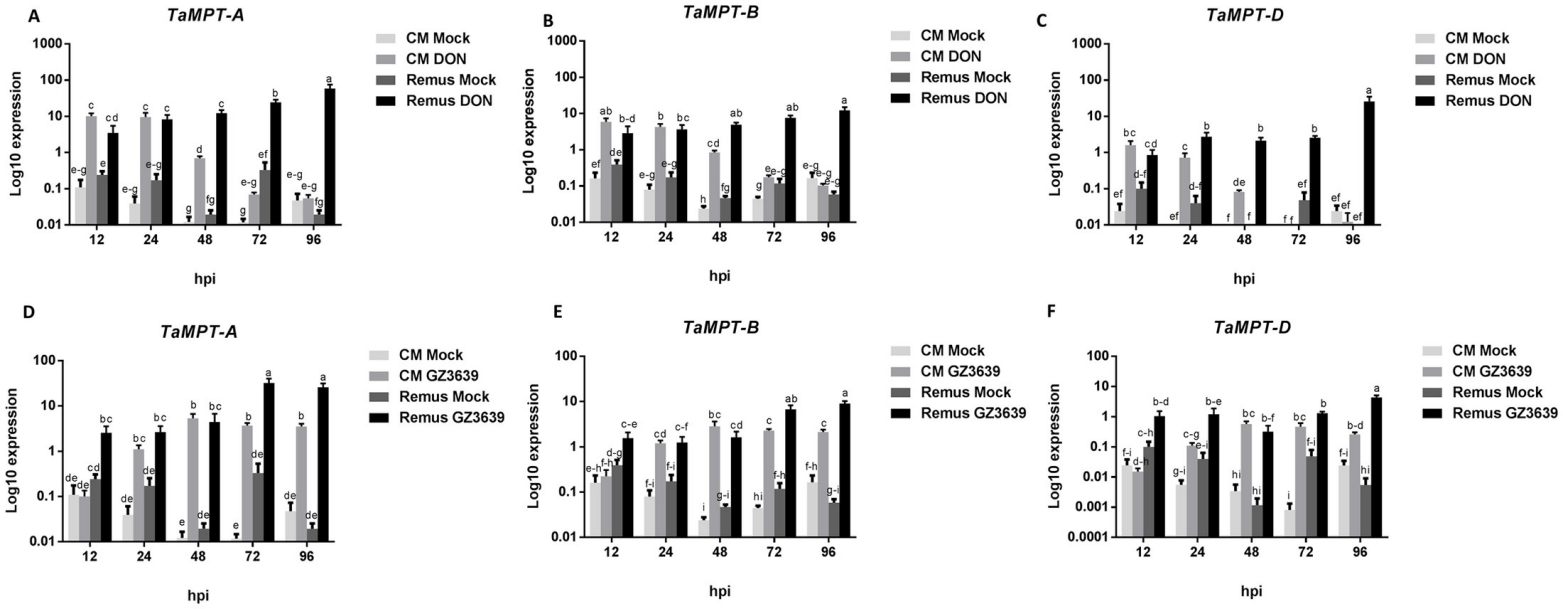
Effect of DON and *F*. *graminearum* on the expression of *TaMPT* homoeologs in spikes of the FHB resistant cv. CM82036 and the susceptible cv. Remus. (A) *TaMPT-A* expression in response to DON. (B) *TaMPT-B* expression in response to DON. (C) *TaMPT-D* expression in response to DON. (D) *TaMPT-A* expression in response to *F*. *graminearum*. (E) *TaMPT-B* expression in response to *F*. *graminearum*. (F) *TaMPT-D* expression in response to *F*. *graminearum*. At mid-anthesis (growth stage 65) [36] two central spikelets of the heads were treated with either DON, *F*. *graminearum* strain GZ3639 (WT) [50] or Tween-20 (mock treatment). harvested at 0, 12, 24, 48, 72, and 96 hours post inoculation, all as previously described [9]. Gene expression was quantified relative to wheat α-tubulin, YLS8 and TaPP2AA3 housekeeping genes (average of [2^^-(CT target- CT α-tubulin)^], [2^^-(CT target- CT YLS8)^] and [2^^-(CT target- CT PP2AA3)^] and is presented on a log_10_ scale on y-axis. Results represent the mean from three biological replicates (n = 6; each sample representing an RNA sample pooled from 8 heads) and bars indicate SEM. Columns with the same letter are, statistically, not significantly different (*P* < 0.05).

At the earlier time points, wheat *TaMPT* genes were generally activated by both DON and FHB in both genotypes, but at later time points, they were activated to a greater extent in the susceptible cv. Remus (Fig 2). Also of note is that all homoeologs were not responsive to FHB in cv. CM82036 until 24h, unlike cv. Remus where they were FHB responsive at 12 hpi. The basal expression of 5A, 5B and 5D homoeologs of *TaMPT* in control (mock) treated tissue was near the detectable limit in both cultivars. In cv. CM82036, DON induction of all the *TaMPT* homoeologs (5A, 5B, and 5D) peaked at 12-24hpi and diminished thereafter, whereas the DON induction of the three variants of the gene in cv. Remus was more constant and generally increased over time, up to 96 hpi (Fig 2A–2C). Peak expression in DON-treated samples was 2-16-fold higher in cv. Remus (at 96hpi) than in cv. CM82036 (at 12 hpi). In terms of their response to *F*. *graminearum* expression of all three variants peaked in cv. CM82036 by 48hpi, with expression slightly, but not significantly (*P >* 0.05), declining thereafter (Fig 2D–2F). In cv. Remus, the expression in response to the pathogen of the 5A variant peaked at 72 hpi whereas that of the 5B and 5D variants peaked at 96 hpi. Peak expression in pathogen-treated tissue was 3–7.5-fold higher in FHB-treated cv. Remus (at 96hpi) as compared to CM82036 (at 48hpi). While all *TaMPT* homoeologs (5A, 5B, and 5D) showed a similar expression pattern in response to DON and wild type *F*. *graminearum* (Fig 2), in both wheat cultivars *TaMPT-A* was more responsive than the other variants to both the pathogen and DON treatment. In the DON-treated samples, at peak expression (12 hpi in cv. CM82036 and 96 hpi in cv. Remus), *TaMPT*-*A* transcript levels were 1.5-6-fold higher than those of either 5B or 5D variants (Fig 2A–2C). Similarly, in *F*. *graminearum*-treated samples, peak expression of the 5A variant in cvs. CM82036 (48 hpi) and Remus (96 hpi) was 2-9-fold higher than that of the other two variants (Fig 2D–2F).

**Fig 3:**
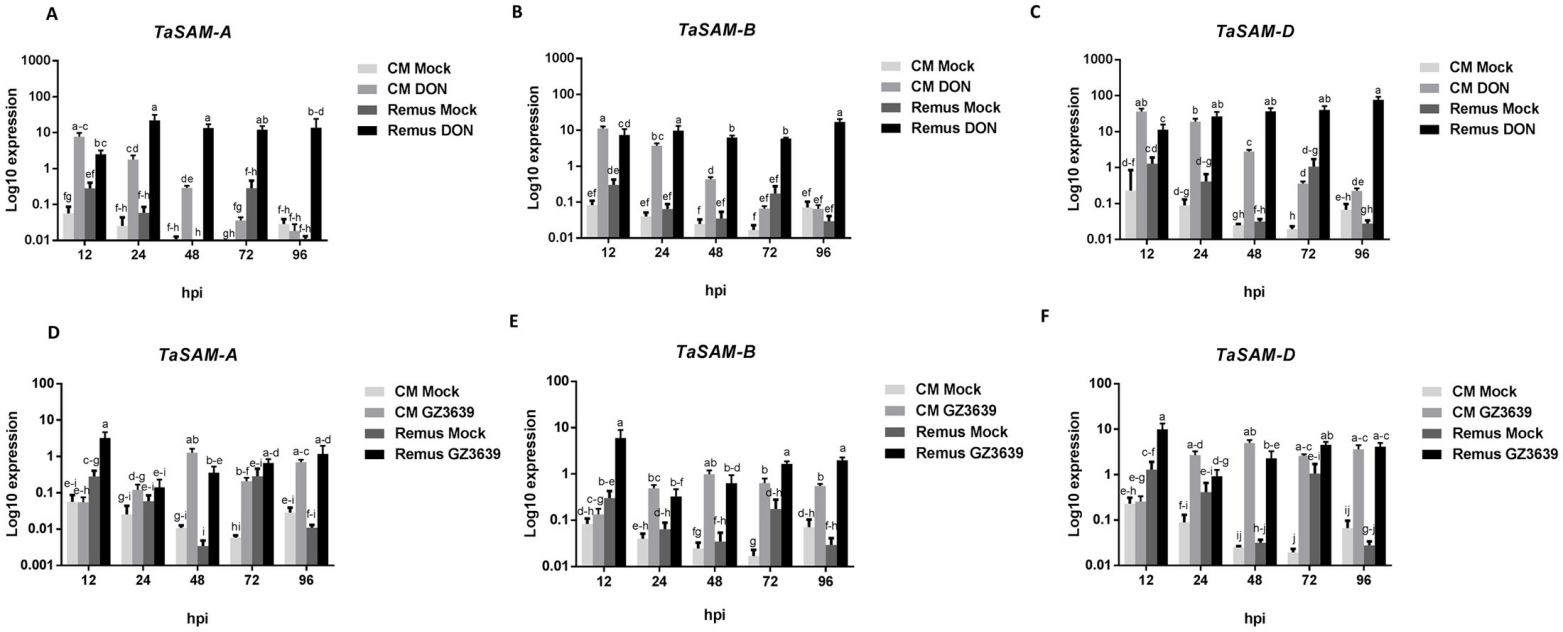
Effect of DON and *F*. *graminearum* on the expression of *TaSAM* homoeologs in spikes of the FHB resistant cv. CM82036 and the susceptible cv. Remus. (A) *TaSAM-A* expression in response to DON. (B) *TaSAM-B* expression in response to DON. (C) *TaSAM-D* expression in response to DON. (D) *TaSAM-A* expression in response to *F*. *graminearum*. (E) *TaSAM-B* expression in response to *F*. *graminearum*. (F) *TaSAM-D* expression in response to *F*. *graminearum*. At mid-anthesis (growth stage 65) [36] two central spikelets of the heads were treated with either DON, *F*. *graminearum* strain GZ3639 (WT) [50] or Tween-20 (mock treatment). harvested at 0, 12, 24, 48, 72, and 96 hours post inoculation, all as previously described [9]. Gene expression was quantified relative to wheat α-tubulin, YLS8, *TaPP2AA3* housekeeping genes (average of [2^^-(CT target- CT α-tubulin)^], [2^^-(CT target- CT YLS8)^] and [2^^-(CT target- CT PP2AA3)^] and is presented on a log_10_ scale on y-axis. Results represent the mean from three biological replicates (n = 6; each sample representing an RNA sample pooled from 8 heads) and bars indicate SEM. Columns with the same letter are, statistically, not significantly different (*P* < 0.05).

*TaSAM* homoeologs were activated earlier in the resistant cv. CM82036 in response to DON, as compared to the susceptible cv. Remus, but the opposite was true for *Fusarium* induction of gene expression. All homoeologs of *TaSAM* (2A, 2B, and 2D) were induced as an early response to DON in both genotypes, peaking at 12-24h in both cultivars, decreasing thereafter in cv. CM82036 but not in cv. Remus (Fig 3A–3C). Peak expression of all three variants in DON-treated samples was 1.5-2-fold higher in cv. Remus (at 96 hpi) than in cv. CM82036 (at 12 hpi). Expression of all three variants was induced by the pathogen at 12 hpi in cv. Remus but not until 24 hpi in cv. CM82036 (Fig 3D–3F). Peak expression in pathogen-treated samples of cv. Remus (at 12 hpi) was 2-6-fold higher than in cv. CM82036 (at 48hpi). *TaSAM-D* was the homoeolog most responsive to the pathogen and DON treatment in both wheat cultivars. Peak expression of the 2D variant in DON-treated samples of cvs. CM82036 (12 hpi) and Remus (96 hpi) was 3–5.5-fold higher than that of either the 2A or 2B homoeologs (Fig 3A–3C). Similarly, in the *Fusarium*-treated samples, peak expression of the 2D variant in cvs CM82036 (48 hpi) and Remus (12 hpi) was 1.7-5-fold higher than that of either the 2A or 2B variants (Fig 3D–3F).

### *TaMPT* and *TaSAM* genes contribute to Type II FHB resistance in wheat

**Fig 4:**
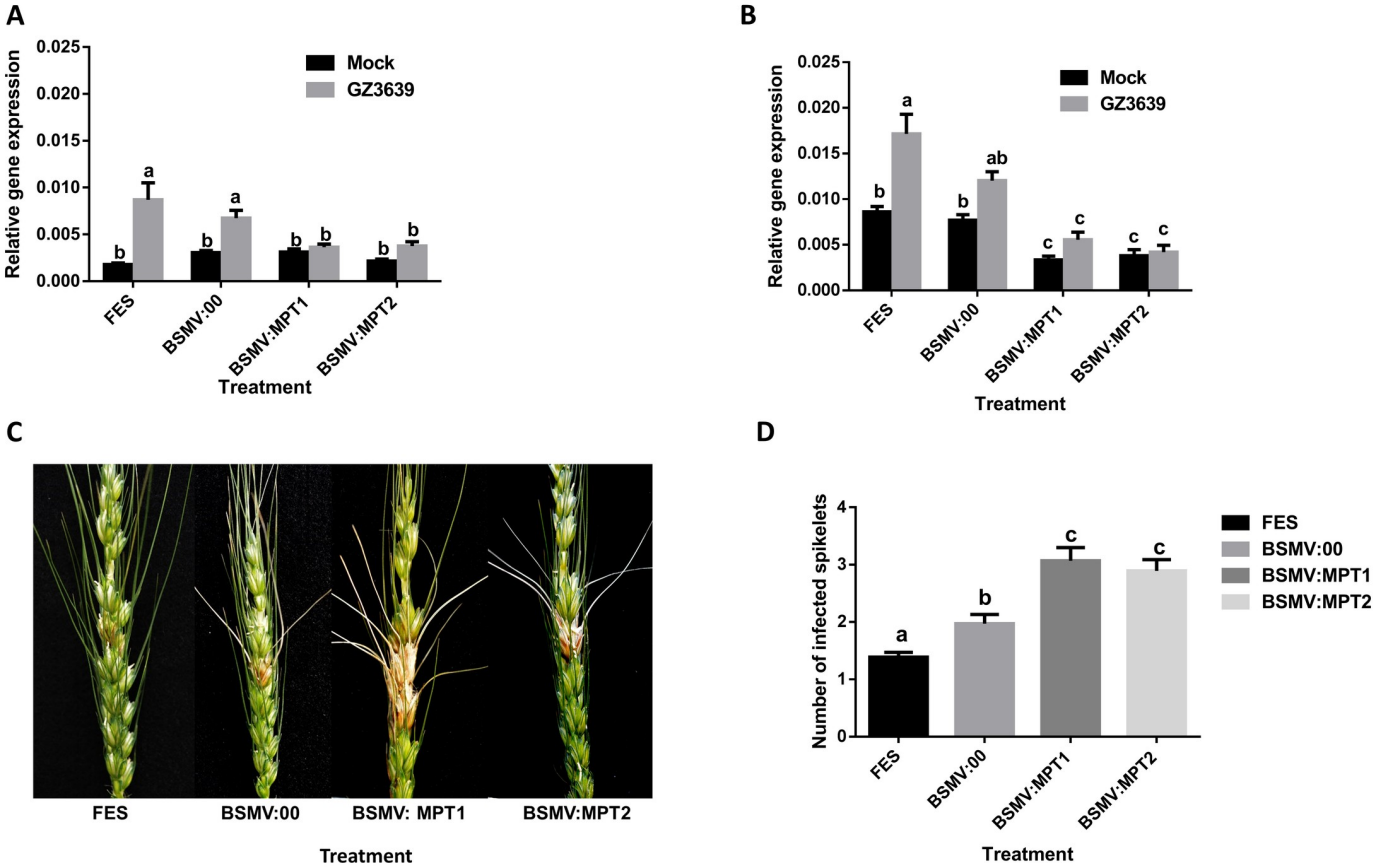
Virus-induced gene silencing of *TaMPT* gene in heads of wheat cv. CM82036. Flag leaves of wheat cv. CM82036 were rub-inoculated at growth stage 47 [36] just before the emergence of the first wheat head with either FES (VIGS buffer), *in vitro* transcribed RNAs from BSMV:00 (empty vector), BSMV: MPT1 or BSMV:MPT2 (constructs targeting *TaMPT*). At mid-anthesis (growth stage 65) [36] two central spikelets of heads were inoculated with either conidia of *F*. *graminearum* strain GZ3639 or Tween-20 (mock treatment), as previously described [9]. After 24h, the third spikelet above the treated spikelets was harvested for gene expression analysis. The specificity of gene silencing was examined using homoeolog-specific primers for (A) *TaMPT-A* or (B) *TaMPT-B* and expression of those genes were quantified by real-time PCR analysis using wheat *α-tubulin*, *YLS8* and *TaPP2AA3* as housekeeping genes (average of [2^^-(CT target- CT *α-tubulin*)^], [2^^-(CT target- CT *YLS8*)^] and [2^^-(CT target- CT *PP2AA3*)^] [48]. Gene expression data represents from the 60 heads per treatment combination (5 bulk RNA from four heads). Disease symptoms were scored at 14 days post-treatment. (C) Images displaying typical disease symptoms at 14 days post-*Fusarium* treatment in gene-silenced as compared to mock (virus) -treated samples. (D) Quantification of the number of diseased spikelets per head in cv. CM82036 at 14 days post-treatment. Disease results represents mean data obtained from 60 heads (20 heads per treatment combination in each of three trials). Bars in graphs indicate standard error of the mean (SEM). Treatments with the same letter are not significantly different (*P >* 0.05).

A VIGS experiment was conducted to determine if *TaMPT* and *TaSAM* genes contribute to Type II FHB resistance in wheat cv. CM82036 (resistance to disease spread from centrally inoculated spikelets). Silencing was independently achieved using two constructs for *TaMPT* (BSMV:MPT1 and BSMV:MPT2) and for *TaSAM* (BSMV:SAM1 and BSMV:SAM2). The constructs specifically targeted all wheat homoeologs located on chromosome 5A, 5B and 5D for *TaMPT* (S3A Fig and S9 Table) and 2A, 2B, and 2D for *TaSAM* (S3B Fig and S9 Table). For the chromosome 5D homoeolog (*TaMPT-D*) the expression was below detectable limits in both control (BSMV:00) and VIGS treated samples (result not shown). In the absence of gene silencing (FES buffer treatment or empty virus BSMV:00 treatment), *TaMPT-A* and *TaMPT-B* were significantly upregulated in response to the fungal pathogen *F*. *graminearum* (*P* < 0.05; Fig 4A and 4B). Silencing of *TaMPT* using either BSMV:MPT1 or BSMV:MPT2 reduced the *Fusarium*-induced transcription of both these genes by 44–65%, as compared to the effect of *Fusarium* on plants treated with the mock virus (BSMV:00) (*P* < 0.05; Fig 4). In the absence of *Fusarium*, low *TaMPT-A* expression was observed, and expression was not significantly different in gene-silenced plants as compared to non-silenced plants. In the absence of the pathogen, *TaMPT-B* expression was significantly lower in gene-silenced plants as compared to non-silenced plants. A potential off-target *TaMPT* gene variant located on chromosome 2 shared 20bp sequence identity with construct BSMV:MPT2, but gene-specific qRT-showed that it was not silenced by either construct, as verified by qRT-PCR (S4 Fig). Plants were visually scored for FHB disease symptoms on wheat heads at 14 and 21 days post-treatment of *Fusarium* for the VIGS experiment. At 14 days post-treatment, plants treated with BSMV:MPT1 and BSMV:MPT2 were 1.5-fold more diseased than BSMV:00 treated plants (*P* < 0.05; Fig 4C and 4D). The average number of infected spikelets in BSMV:00-treated plants was 2.0, while for BSMV:MPT1 and BSMV:MPT2 treatments it was 3.1 and 2.9, respectively. At 21 days post-treatment (S5A and S5B Fig), pink and brown discolouration was observed on diseased spikelets of plants wherein the *TaMPT* gene was silenced. And at this stage, the average number of infected spikelets was 3.0, 5.3 and 4.7 for BSMV:00, BSMV:MPT1 and BSMV:MPT2 treatments, respectively, being >1.6-fold greater for the *TaMPT*-silenced plants (*P* < 0.05; S5A and S5B Fig).

**Fig 5:**
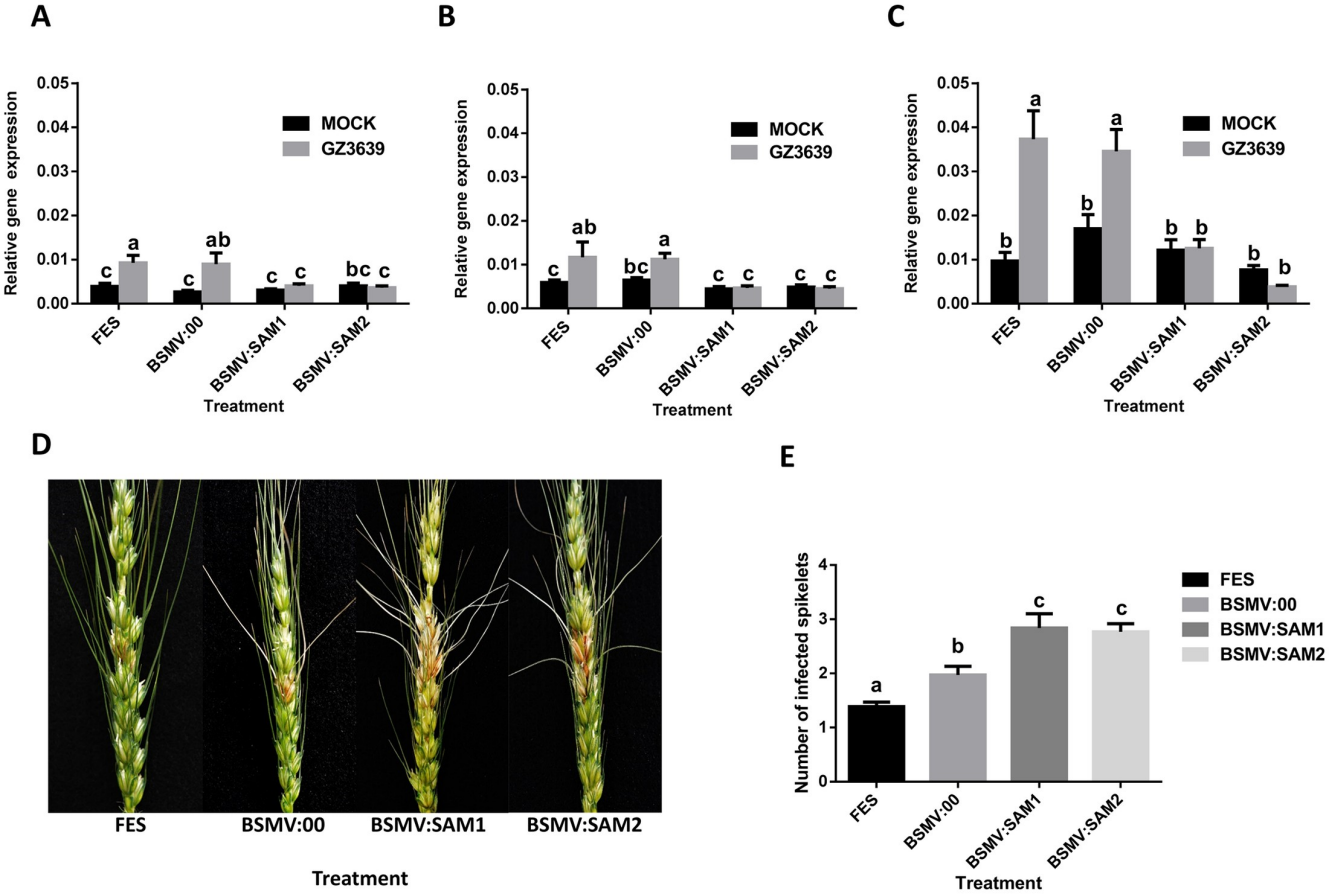
Virus-induced gene silencing of *TaSAM* gene in heads of wheat cv. CM82036. Flag leaves of wheat cv. CM82036 were rub-inoculated at growth stage 47 [36] just before the emergence of the first wheat head with either FES (VIGS buffer), *in vitro* transcribed RNAs from BSMV:00 (empty vector) or BSMV:SAM1 or BSMV:SAM2 (construct targeting *TaSAM*). At mid-anthesis (growth stage 65) [36] two central spikelets of heads were inoculated with either conidia of *F*. *graminearum* strain GZ3639 or 0.02% Tween-20 (mock treatment), as previously described [9]. After 24h, the third spikelet above the treated spikelets was harvested for gene expression analysis. The specificity of gene silencing was examined using homoeolog-specific primers for (A)*TaSAM-A* (B) *TaSAM-B* and (C) *TaSAM-D*, and expression of those genes were quantified by real-time PCR analysis using wheat *α-tubulin*, *YLS8* and *TaPP2AA3* housekeeping genes (average of [2^^-(CT target- CT *α-tubulin*)^], [2^^-(CT target- CT *YLS8*)^] and [2^^-(CT target- CT *PP2AA3*)^] [48]. Gene expression data represents from the 60 heads per treatment combination (5 bulk RNA from four heads). Disease symptoms were scored at 14 days post-treatment. (D) Images displaying typical disease symptoms at 14 days post-*Fusarium* treatment at silenced plants compared to mock (virus) treated samples. (E) Quantification of the number of diseased spikelets per head in cv. CM82036 at 14 days post-treatment. Disease results represents mean data obtained from 60 heads (20 heads per treatment combination in each of three trials). Bars in graphs indicate standard error of the mean (SEM). Treatments with the same letter are not significantly different (*P* > 0.05).

For *TaSAM*, in the absence of gene silencing (FES buffer treatment or empty virus BSMV:00 treatment), all three homoeologs were significantly upregulated in response to *Fusarium* (*P* < 0.05; Fig 5A–5C). In the absence of *Fusarium* treatment, neither BSMV:SAM1 nor BSMV:SAM2 reduced basal *TaSAM* transcript levels, relative to BSMV:00. But, in the presence of *Fusarium*, VIGS reduced the expression of all three homoeologs by 55–89%, as compared to plants treated with the mock virus (BSMV:00) (*P* < 0.05; Fig 5). *Fusarium*-treated heads developed FHB symptoms and, by 14 days post fungal treatment, BSMV:SAM1 and BSMV:SAM2–treated plants showed 1.4-fold more symptoms than BSMV:00 treated plants (*P* < 0.05; Fig 5D and 5E). The average number of infected spikelets per wheat heads at 14 days post-treatment was 2.0, 2.8 and 2.8 for BSMV:00, BSMV:SAM1 and BSMV:SAM2-treated plants, respectively. At 21 days post-treatment, both BSMV:SAM1 and BSMV:SAM2-treated plants showed 1.5-fold more diseased spikelets than BSMV:00-treated plants (*P* < 0.05; S6A and S6B Fig). At this time point, the average number of infected spikelets in BSMV:00, BSMV:SAM1 and BSMV:SAM2-treated plants were 3.0, 4.9 and 4.5, respectively.

### *TaMPT and TaSAM* genes positively influence grain number

**Fig 6:**
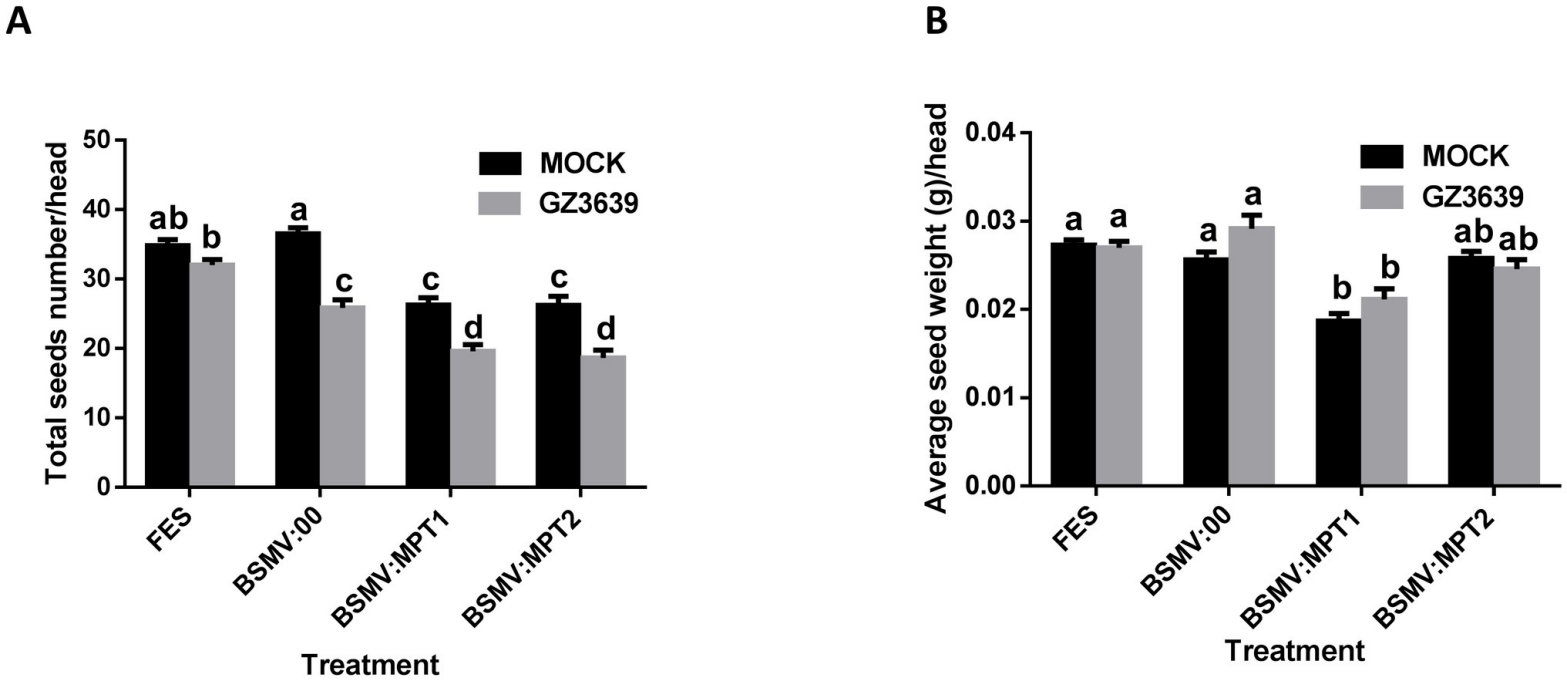
Effect on grain development of virus-induced gene silencing (VIGS) of *TaMPT* genes in wheat heads. Flag leaves of wheat cv. CM82036 were rub-inoculated at growth stage 47 [36] just before the emergence of the first wheat head with either FES (VIGS buffer), or *in vitro* transcribed RNAs from BSMV:00 (empty vector), BSMV:MPT1 or BSMV:MPT2 (construct targeting *TaMPT*). At mid-anthesis (growth stage 65) [36] two central spikelets of heads were inoculated with either conidia of *F*. *graminearum* strain GZ3639 or Tween-20 (mock treatment), as previously described [9]. At harvest, the (A) average seed number per head and (B) average seed weight per head (g) were calculated. Results represent mean data obtained from 60 heads (20 heads per treatment combination in each of three trials). Bars in graphs indicate standard error of the mean (SEM). Treatments with the same letter are not significantly different (*P* > 0.05).

**Fig 7:**
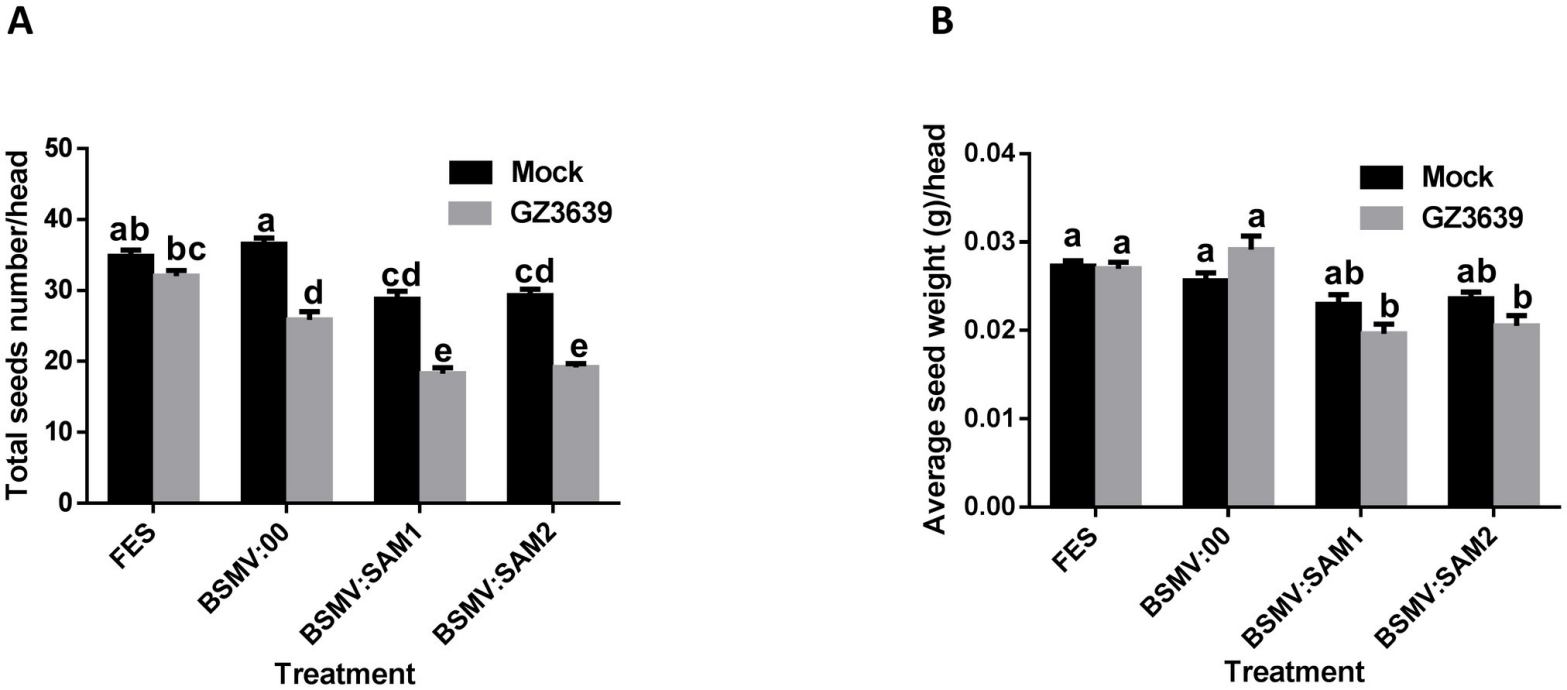
Effect on grain development of virus-induced gene silencing (VIGS) of *TaSAM* genes in wheat heads. Flag leaves of wheat cv. CM82036 were rub-inoculated at growth stage 47 [36] just before the emergence of the first wheat head with representing either FES (VIGS buffer), *in vitro* transcribed RNAs BSMV:00 (empty vector) or BSMV:SAM1 or BSMV:SAM2 (construct targeting *TaSAM*). At mid-anthesis (growth stage 65) [36] two central spikelets of heads were inoculated with either conidia of *F*. *graminearum* strain GZ3639 or 0.02% Tween-20 (mock treatment), as previously described [9]. At harvest, the (A) average seed number per head and (B) average seed weight per head (g) were calculated. Results represent mean data obtained from 60 heads (20 heads per treatment combination in each of three trials). Bars in graphs indicate standard error of the mean (SEM). Treatments with the same letter are not significantly different (*P* > 0.05).

For plants treated with FES (the VIGS buffer), point inoculation of spikelets with *F*. *graminearum* resulted in a small (8%, relative to Tween-20) but insignificant (*P* > 0.05) reduction in grain number, and it did not affect average grain weight (Figs 6 and 7). The effects of *Fusarium* on grain number were greater for plants in which the flag leaf was treated with empty virus BSMV:00 (*P* < 0.05; 29% relative to Tween-20), suggesting that the virus exacerbated the effects of FHB on grain development. For both *TaMPT* and *TaSAM*, the effect of VIGS on grain development were independent of *Fusarium* treatment, as similar effects were observed in both the mock and FHB-treated tissue (Figs 6 and 7). Silencing of *TaMPT* using BSMV:MPT1 in mock and *Fusarium*-inoculated heads resulted in respective reductions of 28 and 24% in grain number. The second construct (BSMV:MPT2) had similar effects to BSMV:MPT1 on grain number (28 and 27% reductions for mock and *Fusarium*-treated heads, relative to BSMV:00; *P <* 0.05) (Fig 6A). In terms of grain weight, BSMV:MPT1 treatment results in a 27% reduction in grain weight in mock and *Fusarium* treated samples (*P* < 0.05), as compared to BSMV:00 treatment (Fig 6B). But construct BSMV:MPT2 treatment did not significantly reduce grain weight in mock and *Fusarium*-treated heads (*P* > 0.05).

VIGS of *TaSAM* using BSMV:SAM1 in mock and *Fusarium*-inoculated heads resulted in respective reductions of 21 and 29% in seed number (*P* < 0.05), as compared to the BSMV:00 treatment (Fig 7A). The second construct (BSMV:SAM2) had similar effects to BSMV:SAM1 on grain number, resulting in a reduction in total grain number of 20 and 26% in mock and *Fusarium*-inoculated heads, respectively (*P* < 0.05). For grain weight, in *Fusarium*-inoculated heads construct BSMV:SAM1 treatment results in a 34% reduction, as compared to BSMV:00 treatment (*P* < 0.05; Fig 7B). In mock-treated samples, the 10% reduction in grain weight in BSMV:SAM1 versus BSMV:00 treated samples was not statistically significant (*P* > 0.05). The second construct (BSMV:SAM2) did not significantly reduce grain weight in mock inoculated heads but resulted in a 29% reduction of grain weight in *Fusarium*-inoculated heads, relative to BSMV:00 (*P <* 0.05) (Fig 7B).

## Discussion

Resistance to FHB is complex and governed by several QTL on wheat chromosomes, indicating that multiple genes affect the resistance [51]. This study highlighted *TaMPT* and *TaSAM* as positive contributors to the wheat defense response against DON mycotoxin and FHB, inhibiting the spread of the disease. In comparing with previous studies and by searching the physical position of QTL-associated markers [52,53] in the cv. Chinese Spring wheat genome (IWGSC v.1.1), we deduced that the physical position of *TaSAM-D* gene is outside (150 kb distal to) the FHB QTL on chromosome 2DS, suggesting that *TaSAM* gene is not directly associated with 2DS QTL [54]. The *TaMPT-A* gene was located within the FHB 5A (*Qfhs*.*ifa-5A)* QTL interval [32]. But in our study, we showed that *TaMPT* contributed to resistance to disease spread rather than the Type I resistance associated with the 5A QTL (resistance to infection). Furthermore, QTL mapping using a DH population of CM82036x Remus [32,55] deduced that *TaMPT-A* was not co-localised within the fine-mapped 5A QTL (*Qfhs*.*ifa-5A*) region (results not shown), suggesting that *TaMPT-A* is not the causal gene within 5A QTL region derived from cv. CM82036 [56].

Gene expression studies showed that DON induced expression of both genes earlier in cv. CM82036, but to a greater extent in cv. Remus. But pathogen up-regulation of both *TaMPT* and *TaSAM* variants was generally as quick, if not quicker, and greater in the susceptible as compared to the resistant cultivar. This is likely reflective of faster DON accumulation in the susceptible as compared to the resistant cultivar. DON production in wheat spikes typically starts at around 36h after fungal inoculation, and high amounts of DON are accumulated between 36–96 hpi [57,58]. Interestingly FHB response of *TaSAM* genes peaked as early as 12hpi; hence it is either responsive to very low toxin levels or to other plant/fungal metabolites that form part of the initial response to FHB disease. There is precedence for the early response of methyltransferases to FHB diseases: the accumulation of methyltransferase gene was earlier and higher at 12 hpi in susceptible cv. Calendonia than FHB resistant cv. Sumai 3 [30].

We hypothesized that genomes would not contribute equally in response to FHB and DON, and consistent with this, we found some variation in the relative expression of the homoeologs with higher expression of *TaMPT-A* and *TaSAM-D*, as compared to their homoeologous counterparts, in both cultivars. In polyploid genomes, homoeologous genes may contribute in an additive manner or may have different expression patterns, giving rise to expression dominance from one or two sub-genomes [59]. Furthermore, homoeologous genes may alternatively been subjected to sub-functionalization. Nussbaumer et al. [59] identified that D subgenome was more abundant and responsive to *F*. *graminearum* than either the A or B subgenome of wheat. RNA-seq studies conducted by Powell et al. [60] identified that B and D homoeologs were more responsive than A homoeolog genes during infection by the fungal pathogen *Fusarium pseudograminearum*, indicating a homoeolog expression bias in hexaploid wheat.

The *TaMPT* genes belong to the phosphate transporter 3 (PHT3) gene family localised in the inner mitochondrial membrane [61]. *TaMPT* is the first *MPT3* gene implicated in FHB resistance. This was validated via VIGS in the FHB resistant wheat cultivar CM82036 wherein it reduced disease spread. It also affected grain development in that gene silencing reduced grain number, while effects of grain weight were observed for one of the two silencing constructs. The closest Arabidopsis ortholog (MPT3, AT5G14040; 77.1% homology) was shown to be responsive to both salt and drought stress. Overexpression of *AtMPT3* increased plant sensitivity to salt stress compared to wild-type plants, suggesting that ATP-dependent pathway are more activated by high *AtMPT3* expression levels under salt stress [62]. Knowledge regarding the biological functions and molecular mechanisms of mitochondrial phosphate transporter in plants is still limited [61]. MPTs, located in the mitochondrial inner membrane, catalyse the transport of phosphate from the cytosol into the mitochondrial matrix where cellular ATP is generated from ADP and phosphate through oxidative phosphorylation [15–17]. Stress alters central metabolic pathways, including protein turnover, reactive oxygen species (ROS) production and changes in redox ratios. These metabolic changes enhances the demand for the fast turnover of ADP to ATP cycle that is mediated by respiratory oxidative phosphorylation [63]. Although ROS is an important signalling molecule in diverse biological processes, excessive amounts are toxic [64]. The *Fusarium* mycotoxin DON stimulates the overproduction of ROS [65]. High amount of H_2_O_2_ accumulation leads to programmed cell death (PCD) [65], which could help the infection process of *F*. *graminearum*. ATP synthesis in mitochondria and higher amounts of phosphate generation is important for the reduction of excessive amounts of ROS. ATP molecules are exported from the mitochondria to the cytoplasm and help to minimise excessive amounts of ROS production [66,67]. Thus, MPT activity might further delay the oxidative burst and reduce FHB disease symptoms and PCD during the fungal infection process.

Silencing of *TaMPT* genes in wheat might result in enhanced damage to mitochondrial proteins, which in turn might have facilitated the accumulation of DON and the overproduction of ROS, thus enabling fungal spread in wheat heads. Many studies have shown that the modulation of mitochondrion-associated proteins disturbed mitochondrial functions that affect both plant growth and development [68]. Overexpressing the *AtMPT3* gene in *Arabidopsis* disturbed the cellular redox homeostasis; transgenic plants accumulated excess amount of H_2_O_2_ and O_2_^-^ levels which lead to PCD and hampered growth and development, suggesting that finely tuned mitochondrion activities are necessary for plant normal growth and development [68]. Tiwari et al. [69] demonstrated that reduced levels of ATP in the mitochondrial cells, due to oxidative stress, resulted in damage to the mitochondrial respiratory chain. It is likely that VIGS of the *TaMPT* gene leads to mitochondrial dysfunction during FHB treatment in the silenced plants. In this study, the observed phenotypes in silencing plants could be explained by the inability of mitochondria to meet the energy demands (ATP synthesis) in cells as a result of reduced Pi transport into mitochondria, which in turn would reduce the electron flux in the mitochondrial membrane and increase ROS formation, leading to more diseased symptoms in the spikelets of silenced plants. Jia et al. [68] reported the induced expression of genes involved in mitochondrial respiratory chain such as ATP synthase and alternative oxidases (*AOX*) in *Arabidopsis MPT3* overexpressing lines using microarray studies. More research is needed to understand if silencing *TaMPT* has effect on other genes involved in mitochondrial respiration in wheat.

It has been suggested that phosphate homeostasis and energy production plays important roles in grain development [18,70]. Wheat mitochondrial phosphate transporter genes (PHT3) were involved in grain development with high expression of *TaPHT3;1* in embryo and rachis, and *TaPHT3;2* in aleurone, suggesting its role in phosphate related homeostasis [18]. Recently, Yu et al. [71] showed that overexpressing tomato mitochondrial phosphate transporter gene (SIMPT3;1) in transgenic rice significantly promoted the uptake of phosphate and increased grain yield. In this study, the reduction in the total number of seeds (due to both VIGS silencing constructs) and weight (due to one of the two VIGS silencing constructs) in *TaMPT*-silenced plants suggests that these genes may have role to play in grain development. Biochemical and genetic studies are needed to decipher the relationship between *TaMPT* and oxidative phosphorylation, ATP synthesis and ROS accumulation and grain development. This will further help us to understand the role of plant mitochondrial phosphate transporters in FHB disease and DON in wheat and other cereals.

*TaSAM* are the first methyltransferase genes functionally characterised for their role in resistance to the spread of FHB disease. But, various microarray and gene expression studies have shown the upregulation of methyltransferase genes in response to *F*. *graminearum* in wheat [28,30]. Cho et al. [28] highlighted a SAM-dependent methyltransferase from a microarray study and the gene was differentially expressed in the resistant cv. Dahongmil and the susceptible cv. Urimil after inoculation with *F*. *graminearum*. Using microarray expression profiling, Long et al. [29] demonstrated that a SAM-methyltransferase gene was upregulated in response to *F*. *graminearum* in a wheat Near isogenic line (NIL) that segregated for a FHB resistance QTL on chromosome 2DL. Recently, AlTaweel et al. [30] found the upregulation of a methyltransferase gene in the presence of *F*. *graminearum* infection in the FHB resistance cv. Sumai 3 and the susceptible cv. Caledonia, and they suggested that the methyltransferase may be involved in the response to oxidative stress. In this study, a DON and FHB-responsive *TaSAM* gene from the SAM-methyltransferase superfamily was shown to contribute to FHB resistance in wheat. The *Arabidopsis* ortholog (*AT2G41380)*, which shares 53% homology with *TaSAM-2D*, is responsive to ROS and acts against oxidative stress [72–74]. Further experiments are needed to determine the role, if any, of *TaSAM* gene against oxidative stress in wheat. In rice, knockdown of *OsSAMS*1, 2 and 3 using RNA interference resulted in late flowering, dwarfism, and reduced fertility in transgenic plants, suggesting a putative role of this gene in histone H3K4me3 and DNA methylation. Moreover, they proposed that SAM deficiency or transport reduces SAM-dependent methyltransferase activities, leading to hypo methylation in plants [75]. Despite the role of methyltransferase in methylation and biosynthesis, to our knowledge, SAM-methyltransferase are rarely reported to be involved in grain yield and development in wheat. Previously, a study has shown that disruption of a methyltransferase gene was associated with reduced grain yield in rice. Hong et al. [76] demonstrated that disruption of the rice methyltransferase gene *OsMTS1* resulted in premature leaf senescence, a low rate of photosynthesis, accumulation of ROS and grain yield reduction. The reduced grain number and weight in *TaSAM* silenced plants in our study suggested that it may have role in wheat yield components.

In conclusion, VIGS studies showed that *TaMPT* and *TaSAM* genes positively contributed to FHB resistance in wheat, most likely indirectly rather than through direct effects on the pathogen or DON. The results of VIGS study suggests that both *TaMPT* and *TaSAM* genes have potential for enhancing FHB resistance and augmenting grain development in wheat. Thus, *TaMPT* and *TaSAM* add to the relatively short list of FHB resistance genes that can be used to engineer crops with improved FHB resistance and yield performance. More detailed investigations of *TaMPT* and *TaSAM* genes and their pathways in wheat will extend our understanding of FHB resistance, and the functional roles and mechanisms of *TaMPT* and *TaSAM*.

## Supporting information

S1 Fig Protein alignment and conserved domain identification of *TaMPT-A* from wheat cv. CM82036 with cv. Remus and variants from wheat cv. Chinese spring. (TIF)

S2 Fig Protein alignment and conserved domain identification of *TaSAM-D* from wheat cv. CM82036 with cv. Remus and variants from wheat cv. Chinese spring (CS). (TIF)

S3 Fig The relative position of the non-overlapping fragments targeted for gene silencing (two fragments: VIGS 1 and 2) and for qRT-PCR validation of VIGS efficacy. (A) *TaMPT* (chromosomes 5A, 5B and 5D). (B) *TaSAM* (chromosomes 2A, 2B and 2D).
(TIF)

S4 Fig Expression validation of virus-induced gene silencing (VIGS) of mitochondrial phosphate transporter gene (*TaMPT*) in wheat on the transcription of chromosome 2 (off target genes). Flag leaves of wheat cv. CM82036 were rub-inoculated at growth stage 47 [36] just before the emergence of the first wheat head with representing either FES (VIGS buffer), *in vitro* transcribed RNAs BSMV:00 (empty vector) or BSMV: MPT1 or BSMV: MPT2 (construct targeting *TaMPT*). At mid-anthesis (growth stage 65) [36] two central spikelets of heads were inoculated with either conidia of *F*. *graminearum* strain GZ3639 or Tween-20 (mock treatment), as previously described [9]. After 24h, the third spikelet above the treated spikelets was harvested for gene expression analysis. The expression of *TaMPT* on chromosome 2 was quantified by real-time PCR analysis using wheat *α-tubulin*, *YLS8* and *TaPP2AA3* housekeeping genes (average of [2^^-(CT target- CT *α-tubulin*)^], [2^^-(CT target- CT *YLS8*)^] and [2^^-(CT target- CT *PP2AA3*)^] [48]. Gene expression data represents from the 60 heads per treatment combination (5 bulk RNA from four heads). Bars in graphs indicate standard error of the mean (SEM). Treatments with the same letter are not significantly different (*P* > 0.05).
(TIF)

S5 Fig Virus-induced gene silencing of *TaMPT* gene in wheat. Flag leaves of wheat cv. CM82036 were rub-inoculated at growth stage 47 [36] just before the emergence of the first wheat head with representing either FES (VIGS buffer), *in vitro* transcribed RNAs BSMV:00 (empty vector) or BSMV: MPT1 or BSMV:MPT2 (construct targeting *TaMPT*). At mid-anthesis (growth stage 65) [36] two central spikelets of heads were inoculated with either conidia of *F*. *graminearum* strain GZ3639 or 0.02% Tween-20 (mock treatment), as previously described [9]. Disease symptoms were scored at 21 days post-treatment. (A) Images displaying typical disease symptoms at 21 days post-*Fusarium* treatment at silenced plants compared to mock (virus) treated samples. B) Quantification of the number of diseased spikelets per head in cv. CM82036 at 21 days post-treatment. Disease results represents mean data obtained from 60 heads (20 heads per treatment combination in each of three trials). Bars in graphs indicate standard error of the mean (SEM). Treatments with the same letter are not significantly different (*P* > 0.05).
(TIF)

S6 Fig Virus-induced gene silencing (VIGS) of *TaSAM* gene in wheat. Flag leaves of wheat cv. CM82036 were rub-inoculated at growth stage 47 [36] just before the emergence of the first wheat head with representing either FES (VIGS buffer), *in vitro* transcribed RNAs BSMV:00 (empty vector) or BSMV: SAM1 or BSMV:SAM2 (construct targeting *TaSAM*). At mid-anthesis (growth stage 65) [36] two central spikelets of heads were inoculated with either conidia of *F*. *graminearum* strain GZ3639 or 0.02% Tween-20 (mock treatment), as previously described [9]. Disease symptoms were scored at 21 days post-treatment. (A) Images displaying typical disease symptoms at 21 days post-*Fusarium* treatment at silenced plants compared to mock (virus) treated samples. (B) Quantification of the number of diseased spikelets per head in cv. CM82036 at 21 days post-treatment. Disease results represents mean data obtained from 60 heads (20 heads per treatment combination in each of three trials). Bars in graphs indicate standard error of the mean (SEM). Treatments with the same letter are not significantly different (*P* > 0.05).
(TIF)

S1 Table Experimental design for plant experiments. (DOCX)

S2 Table List of primers used in this study. (DOCX)

S3 Table DNA sequence similarity between the conserved domains of *TaMPT-A* from wheat cv. CM82036 and homoeologs from cvs. Remus and Chinese spring. (DOCX)

S4 Table Protein sequence similarity of *TaMPT-A* from wheat cv. CM82036 with *TaMPT-A* Remus with 5A, 5B, and 5D homoeologs of Chinese spring. (DOCX)

S5 Table Domain position and sub-cellular localisation of *TaMPT* gene and their homoeologs. (DOCX)

S6 Table DNA sequence similarity between the conserved domains of *TaSAM-D* from wheat cv. CM82036 and homoeologs from cvs. Remus and Chinese spring. (DOCX)

S7 Table Protein sequence similarity of *TaSAM-D* from wheat cv. CM82036 with *TaSAM-D* Remus, 2A, 2B, and 2D homoeologs of Chinese spring. (DOCX)

S8 Table Domain position and sub-cellular localisation of *TaSAM* gene and their homoeologs. (DOCX)

S9 Table Specificity of the mitochondrial phosphate transporter (*TaMPT*) and methyltransferase (*TaSAM*) construct used for VIGS. (DOCX)

## References

[1] Dweba C, Figlan S, Shimelis H, Motaung T, Sydenham S, Mwadzingeni L, et al. Fusarium head blight of wheat: Pathogenesis and control strategies. Crop Protection. 2017;91:114–22.

[2] Desjardins AE, Proctor RH, Bai G, McCormick SP, Shaner G, Buechley G, et al. Reduced virulence of trichothecene-nonproducing mutants of Gibberella zeae in wheat field tests. MPMI-Molecular Plant Microbe Interactions. 1996;9(9):775–81.

[3] Gunupuru L, Perochon A, Doohan F. Deoxynivalenol resistance as a component of FHB resistance. Tropical Plant Pathology. 2017:1–9.

[4] Lulin M, Yi S, Aizhong C, Zengjun Q, Liping X, Peidu C, et al. Molecular cloning and characterization of an up-regulated UDP-glucosyltransferase gene induced by DON from Triticum aestivum L. cv. Wangshuibai. Molecular biology reports. 2010;37(2):785. doi: 10.1007/s11033-009-9606-3 19585272

[5] Zuo D, Yi S, Liu R, Li H, Qu B, Huang T, et al. A deoxynivalenol-activated methionyl-tRNA synthetase gene from wheat encodes a nuclear localized protein and protects plants against Fusarium pathogens and mycotoxins. Phytopathology. 2016;(ja). doi: 10.1094/PHYTO-12-15-0327-R 26882849

[6] Foroud N, Ouellet T, Laroche A, Oosterveen B, Jordan M, Ellis B, et al. Differential transcriptome analyses of three wheat genotypes reveal different host response pathways associated with Fusarium head blight and trichothecene resistance. Plant Pathology. 2012;61(2):296–314.

[7] Lemmens M, Scholz U, Berthiller F, Dall’Asta C, Koutnik A, Schuhmacher R, et al. The ability to detoxify the mycotoxin deoxynivalenol colocalizes with a major quantitative trait locus for Fusarium head blight resistance in wheat. Molecular Plant-Microbe Interactions. 2005;18(12):1318–24. doi: 10.1094/MPMI-18-1318 16478051

[8] Li X, Shin S, Heinen S, Dill-Macky R, Berthiller F, Nersesian N, et al. Transgenic wheat expressing a barley UDP-glucosyltransferase detoxifies deoxynivalenol and provides high levels of resistance to Fusarium graminearum. Molecular Plant-Microbe Interactions. 2015;28(11):1237–46. doi: 10.1094/MPMI-03-15-0062-R 26214711

[9] Gunupuru LR, Arunachalam C, Malla KB, Kahla A, Perochon A, Jia J, et al. A wheat cytochrome P450 enhances both resistance to deoxynivalenol and grain yield. PloS one. 2018;13(10):e0204992. doi: 10.1371/journal.pone.0204992 30312356 PMC6185721

[10] Perochon A, Jianguang J, Kahla A, Arunachalam C, Scofield SR, Bowden S, et al. TaFROG encodes a Pooideae orphan protein that interacts with SnRK1 and enhances resistance to the mycotoxigenic fungus Fusarium graminearum. Plant physiology. 2015;169(4):2895–906. 26508775 10.1104/pp.15.01056 PMC4677899

[11] Walter S, Kahla A, Arunachalam C, Perochon A, Khan MR, Scofield SR, et al. A wheat ABC transporter contributes to both grain formation and mycotoxin tolerance. Journal of experimental botany. 2015;66(9):2583–93. 25732534 10.1093/jxb/erv048 PMC4986867

[12] Perochon A, Váry Z, Malla KB, Halford NG, Paul MJ, Doohan FM. The wheat SnRK1α family and its contribution to Fusarium toxin tolerance. Plant Science. 2019;288:110217. doi: 10.1016/j.plantsci.2019.110217 31521211

[13] Walter S, Brennan JM, Arunachalam C, Ansari KI, Hu X, Khan MR, et al. Components of the gene network associated with genotype-dependent response of wheat to the Fusarium mycotoxin deoxynivalenol. Functional & integrative genomics. 2008;8(4):421–7. doi: 10.1007/s10142-008-0089-4 18592282

[14] Boddu J, Cho S, Muehlbauer GJ. Transcriptome analysis of trichothecene-induced gene expression in barley. Molecular Plant-Microbe Interactions. 2007;20(11):1364–75. doi: 10.1094/MPMI-20-11-1364 17977148

[15] Hamel P, Saint-Georges Y, De Pinto B, Lachacinski N, Altamura N, Dujardin G. Redundancy in the function of mitochondrial phosphate transport in Saccharomyces cerevisiae and Arabidopsis thaliana. Molecular microbiology. 2004;51(2):307–17. doi: 10.1046/j.1365-2958.2003.03810.x 14756774

[16] Haferkamp I. The diverse members of the mitochondrial carrier family in plants. FEBS letters. 2007;581(12):2375–9. doi: 10.1016/j.febslet.2007.02.020 17321523

[17] Takabatake R, Hata S, Taniguchi M, Kouchi H, Sugiyama T, Izui K. Isolation and characterization of cDNAs encoding mitochondrial phosphate transporters in soybean, maize, rice, and Arabidopsis. Plant molecular biology. 1999;40(3):479–86. doi: 10.1023/a:1006285009435 10437831

[18] Shukla V, Kaur M, Aggarwal S, Bhati KK, Kaur J, Mantri S, et al. Tissue specific transcript profiling of wheat phosphate transporter genes and its association with phosphate allocation in grains. Scientific reports. 2016;6:39293. doi: 10.1038/srep39293 27995999 PMC5172359

[19] Golkari S, Gilbert J, Prashar S, Procunier JD. Microarray analysis of Fusarium graminearum-induced wheat genes: identification of organ-specific and differentially expressed genes. Plant Biotechnology Journal. 2007;5(1):38–49. doi: 10.1111/j.1467-7652.2006.00213.x 17207255

[20] Yu X, Wang X, Wang C, Chen X, Qu Z, Yu X, et al. Wheat defense genes in fungal (Puccinia striiformis) infection. Functional & Integrative Genomics. 2010;10(2):227–39. doi: 10.1007/s10142-010-0161-8 20186453

[21] Xin M, Wang X, Peng H, Yao Y, Xie C, Han Y, et al. Transcriptome comparison of susceptible and resistant wheat in response to powdery mildew infection. Genomics, proteomics & bioinformatics. 2012;10(2):94–106. doi: 10.1016/j.gpb.2012.05.002 22768983 PMC5054165

[22] Zanke CD, Rodemann B, Ling J, Muqaddasi QH, Plieske J, Polley A, et al. Genome-wide association mapping of resistance to eyespot disease (Pseudocercosporella herpotrichoides) in European winter wheat (Triticum aestivum L.) and fine-mapping of Pch1. Theoretical and Applied Genetics. 2017;130(3):505–14. doi: 10.1007/s00122-016-2830-z 27866227

[23] Zhang J, Zheng YG. SAM/SAH analogs as versatile tools for SAM-dependent methyltransferases. ACS chemical biology. 2015;11(3):583–97. doi: 10.1021/acschembio.5b00812 26540123 PMC5772741

[24] Martin JL, McMillan FM. SAM (dependent) I AM: the S-adenosylmethionine-dependent methyltransferase fold. Current opinion in structural biology. 2002;12(6):783–93. doi: 10.1016/s0959-440x(02)00391-3 12504684

[25] Gunnaiah R, Kushalappa AC, Duggavathi R, Fox S, Somers DJ. Integrated metabolo-proteomic approach to decipher the mechanisms by which wheat QTL (Fhb1) contributes to resistance against Fusarium graminearum. PloS one. 2012;7(7):e40695. doi: 10.1371/journal.pone.0040695 22866179 PMC3398977

[26] Schweiger W, Steiner B, Vautrin S, Nussbaumer T, Siegwart G, Zamini M, et al. Suppressed recombination and unique candidate genes in the divergent haplotype encoding Fhb1, a major Fusarium head blight resistance locus in wheat. Theoretical and Applied Genetics. 2016:1–17. 10.1007/s00122-016-2727-x PMC4943984 27174222

[27] Li G, Zhou J, Jia H, Gao Z, Fan M, Luo Y, et al. Mutation of a histidine-rich calcium-binding-protein gene in wheat confers resistance to Fusarium head blight. Nature genetics. 2019:1. 10.1038/s41588-019-0426-7 31182810

[28] Cho S-H, Lee J, Jung K-H, Lee Y-W, Park J-C, Paek N-C. Genome-wide analysis of genes induced by Fusarium graminearum infection in resistant and susceptible wheat cultivars. Journal of Plant Biology. 2012;55(1):64–72.

[29] Long X, Balcerzak M, Gulden S, Cao W, Fedak G, Wei Y-M, et al. Expression profiling identifies differentially expressed genes associated with the fusarium head blight resistance QTL 2DL from the wheat variety Wuhan-1. Physiological and Molecular Plant Pathology. 2015;90:1–11.

[30] AlTaweel K, Amarasinghe CC, Brûlé-Babel AL, Fernando WD. Gene expression analysis of host–pathogen interaction between wheat and Fusarium graminearum. European Journal of Plant Pathology. 2017;3(148):617–29.

[31] Buerstmayr M, Steiner B, Wagner C, Schwarz P, Brugger K, Barabaschi D, et al. High-resolution mapping of the pericentromeric region on wheat chromosome arm 5 AS harbouring the Fusarium head blight resistance QTL Qfhs. ifa-5A. Plant biotechnology journal. 2018;16(5):1046–56. doi: 10.1111/pbi.12850 29024288 PMC5902775

[32] Buerstmayr H, Steiner B, Hartl L, Griesser M, Angerer N, Lengauer D, et al. Molecular mapping of QTLs for Fusarium head blight resistance in spring wheat. II. Resistance to fungal penetration and spread. Theoretical and Applied Genetics. 2003;107(3):503–8. doi: 10.1007/s00122-003-1272-6 12768240

[33] Bai G-H, Desjardins A, Plattner R. Deoxynivalenol-nonproducing Fusarium graminearum causes initial infection, but does not cause DiseaseSpread in wheat spikes. Mycopathologia. 2002;153(2):91–8. doi: 10.1023/a:1014419323550 12000132

[34] Bai G-H, Shaner G. Variation in Fusarium graminearum and cultivar resistance to wheat scab. Plant disease. 1996.

[35] Brennan J, Egan D, Cooke B, Doohan F. Effect of temperature on head blight of wheat caused by Fusarium culmorum and F. graminearum. Plant Pathology. 2005;54(2):156–60.

[36] Zadoks JC, Chang TT, Konzak CF. A decimal code for the growth stages of cereals. Weed research. 1974;14(6):415–21.

[37] Proctor RH, Hohn TM, McCormick SP. Reduced virulence of Gibberella zeae caused by disruption of a trichthecine toxin biosynthetic gene. MPMI-Molecular Plant Microbe Interactions. 1995;8(4):593–601. 8589414 10.1094/mpmi-8-0593

[38] Ansari KI, Walter S, Brennan JM, Lemmens M, Kessans S, McGahern A, et al. Retrotransposon and gene activation in wheat in response to mycotoxigenic and non-mycotoxigenic-associated Fusarium stress. Theoretical and Applied Genetics. 2007;114(5):927–37. doi: 10.1007/s00122-006-0490-0 17256175

[39] Wang Y, Tiwari VK, Rawat N, Gill BS, Huo N, You FM, et al. GSP: a web-based platform for designing genome-specific primers in polyploids. Bioinformatics. 2016;32(15):2382–3. 27153733 10.1093/bioinformatics/btw134

[40] Kersey PJ, Allen JE, Armean I, Boddu S, Bolt BJ, Carvalho-Silva D, et al. Ensembl Genomes 2016: more genomes, more complexity. Nucleic acids research. 2015;44(D1):D574–D80. 26578574 10.1093/nar/gkv1209 PMC4702859

[41] Kumar S, Stecher G, Tamura K. MEGA7: molecular evolutionary genetics analysis version 7.0 for bigger datasets. Molecular biology and evolution. 2016;33(7):1870–4. 27004904 10.1093/molbev/msw054 PMC8210823

[42] Blum T, Briesemeister S, Kohlbacher O. MultiLoc2: integrating phylogeny and Gene Ontology terms improves subcellular protein localization prediction. BMC bioinformatics. 2009;10(1):274. doi: 10.1186/1471-2105-10-274 19723330 PMC2745392

[43] Finn RD, Attwood TK, Babbitt PC, Bateman A, Bork P, Bridge AJ, et al. InterPro in 2017—beyond protein family and domain annotations. Nucleic acids research. 2016;45(D1):D190–D9. 27899635 10.1093/nar/gkw1107 PMC5210578

[44] Holzberg S, Brosio P, Gross C, Pogue GP. Barley stripe mosaic virus-induced gene silencing in a monocot plant. The Plant Journal. 2002;30(3):315–27. doi: 10.1046/j.1365-313x.2002.01291.x 12000679

[45] Scofield SR, Huang L, Brandt AS, Gill BS. Development of a virus-induced gene-silencing system for hexaploid wheat and its use in functional analysis of the Lr21-mediated leaf rust resistance pathway. Plant Physiology. 2005;138(4):2165–73. 16024691 10.1104/pp.105.061861 PMC1183404

[46] Wang Y, Tiwari VK, Rawat N, Gill BS, Huo N, You FM, et al. GSP: a web-based platform for designing genome-specific primers in polyploids. Bioinformatics. 2016;32(15):2382–3. Epub 2016/05/07. . 27153733 10.1093/bioinformatics/btw134

[47] Xiang Y, Song M, Wei Z, Tong J, Zhang L, Xiao L, et al. A jacalin-related lectin-like gene in wheat is a component of the plant defence system. Journal of experimental botany. 2011;62(15):5471–83. 21862481 10.1093/jxb/err226 PMC3223046

[48] Livak KJ, Schmittgen TD. Analysis of relative gene expression data using real-time quantitative PCR and the 2− ΔΔCT method. methods. 2001;25(4):402–8. doi: 10.1006/meth.2001.1262 11846609

[49] Zuckerkandl E, Pauling L. Evolutionary divergence and convergence in proteins. Evolving genes and proteins: Elsevier; 1965. p. 97–166.

[50] Proctor R, Hohn T, McCormick S. Main content area Reduced virulence of Gibberella zeae caused by disruption of a trichothecene toxin biosynthetic gene. Molecular plant-microbe interactions. 1995;8(4):593–601. 8589414 10.1094/mpmi-8-0593

[51] Buerstmayr H, Ban T, Anderson JA. QTL mapping and marker-assisted selection for Fusarium head blight resistance in wheat: a review. Plant breeding. 2009;128(1):1–26.

[52] Jia H, Zhou J, Xue S, Li G, Yan H, Ran C, et al. A journey to understand wheat fusarium head blight resistance in the Chinese wheat landrace Wangshuibai. The Crop Journal. 2017.

[53] Steiner B, Lemmens M, Griesser M, Scholz U, Schondelmaier J, Buerstmayr H. Molecular mapping of resistance to Fusarium head blight in the spring wheat cultivar Frontana. Theoretical and Applied Genetics. 2004;109(1):215–24. doi: 10.1007/s00122-004-1620-1 14997302

[54] Shen X, Zhou M, Lu W, Ohm H. Detection of Fusarium head blight resistance QTL in a wheat population using bulked segregant analysis. Theoretical and Applied Genetics. 2003;106(6):1041–7. doi: 10.1007/s00122-002-1133-8 12671752

[55] Buerstmayr H, Lemmens M, Hartl L, Doldi L, Steiner B, Stierschneider M, et al. Molecular mapping of QTLs for Fusarium head blight resistance in spring wheat. I. Resistance to fungal spread (Type II resistance). Theoretical and Applied Genetics. 2002;104(1):84–91. doi: 10.1007/s001220200009 12579431

[56] Steiner B, Buerstmayr M, Wagner C, Danler A, Eshonkulov B, Ehn M, et al. Fine-mapping of the Fusarium head blight resistance QTL Qfhs. ifa-5A identifies two resistance QTL associated with anther extrusion. Theoretical and Applied Genetics. 2019:1–15. doi: 10.1007/s00122-019-03336-x 30949717 PMC6588648

[57] Chen L, Song Y, Xu Y. Variation in the concentrations of deoxynivalenol in the spikes of winter wheat infected by Fusarium graminearum Schw. Acta phytopathologica sinica. 1996;26(1):25–8.

[58] Savard ME, Sinha RC, Lloyd Seaman W, Fedak G. Sequential distribution of the mycotoxin deoxynivalenol in wheat spikes after inoculation with Fusarium graminearum. Canadian Journal of Plant Pathology. 2000;22(3):280–5.

[59] Nussbaumer T, Warth B, Sharma S, Ametz C, Bueschl C, Parich A, et al. Joint transcriptomic and metabolomic analyses reveal changes in the primary metabolism and imbalances in the subgenome orchestration in the bread wheat molecular response to Fusarium graminearum. G3: Genes, Genomes, Genetics. 2015:g3. 115.021550. 26438291 10.1534/g3.115.021550 PMC4683631

[60] Powell JJ, Fitzgerald TL, Stiller J, Berkman PJ, Gardiner DM, Manners JM, et al. The defence-associated transcriptome of hexaploid wheat displays homoeolog expression and induction bias. Plant biotechnology journal. 2017;15(4):533–43. doi: 10.1111/pbi.12651 27735125 PMC5362679

[61] Wang D, Lv S, Jiang P, Li Y. Roles, regulation, and agricultural application of plant phosphate transporters. Frontiers in plant science. 2017;8:817. doi: 10.3389/fpls.2017.00817 28572810 PMC5435767

[62] Zhu W, Miao Q, Sun D, Yang G, Wu C, Huang J, et al. The mitochondrial phosphate transporters modulate plant responses to salt stress via affecting ATP and gibberellin metabolism in Arabidopsis thaliana. PLoS One. 2012;7(8):e43530. doi: 10.1371/journal.pone.0043530 22937061 PMC3427375

[63] Jacoby RP, Li L, Huang S, Pong Lee C, Millar AH, Taylor NL. Mitochondrial Composition, Function and Stress Response in Plants F. Journal of integrative plant biology. 2012;54(11):887–906. doi: 10.1111/j.1744-7909.2012.01177.x 23046139

[64] Apel K, Hirt H. Reactive oxygen species: metabolism, oxidative stress, and signal transduction. Annu Rev Plant Biol. 2004;55:373–99. doi: 10.1146/annurev.arplant.55.031903.141701 15377225

[65] Desmond OJ, Manners JM, Stephens AE, Maclean DJ, Schenk PM, Gardiner DM, et al. The Fusarium mycotoxin deoxynivalenol elicits hydrogen peroxide production, programmed cell death and defence responses in wheat. Molecular Plant Pathology. 2008;9(4):435–45. doi: 10.1111/j.1364-3703.2008.00475.x 18705859 PMC6640518

[66] Noctor G, De Paepe R, Foyer CH. Mitochondrial redox biology and homeostasis in plants. Trends in plant science. 2007;12(3):125–34. doi: 10.1016/j.tplants.2007.01.005 17293156

[67] Jones DP. Disruption of mitochondrial redox circuitry in oxidative stress. Chemico-biological interactions. 2006;163(1–2):38–53. doi: 10.1016/j.cbi.2006.07.008 16970935

[68] Jia F, Wan X, Zhu W, Sun D, Zheng C, Liu P, et al. Overexpression of mitochondrial phosphate transporter 3 severely hampers plant development through regulating mitochondrial function in Arabidopsis. PloS one. 2015;10(6):e0129717. doi: 10.1371/journal.pone.0129717 26076137 PMC4468087

[69] Tiwari BS, Belenghi B, Levine A. Oxidative stress increased respiration and generation of reactive oxygen species, resulting in ATP depletion, opening of mitochondrial permeability transition, and programmed cell death. Plant physiology. 2002;128(4):1271–81. 11950976 10.1104/pp.010999 PMC154255

[70] Cao H, He M, Zhu C, Yuan L, Dong L, Bian Y, et al. Distinct metabolic changes between wheat embryo and endosperm during grain development revealed by 2D-DIGE-based integrative proteome analysis. Proteomics. 2016;16(10):1515–36. doi: 10.1002/pmic.201500371 26968330

[71] Yu G-h, Huang S-c, He R, Li Y-z, Cheng X-g. Transgenic Rice Overexperessing a Tomato Mitochondrial Phosphate Transporter, SlMPT3; 1, Promotes Phosphate Uptake and Increases Grain Yield. Journal of Plant Biology. 2018;61(6):383–400.

[72] Rosenwasser S, Rot I, Sollner E, Meyer AJ, Smith Y, Leviatan N, et al. Organelles contribute differentially to ROS-related events during extended darkness. Plant physiology. 2011:pp. 110.169797. 10.1104/pp.110.169797 PMC3091045 21372201

[73] Benina M, Ribeiro DM, Gechev TS, MUELLER-ROEBER B, Schippers JH. A cell type-specific view on the translation of mRNA s from ROS-responsive genes upon paraquat treatment of A rabidopsis thaliana leaves. Plant, cell & environment. 2015;38(2):349–63. 10.1111/pce.12355 24738758

[74] Gechev T, Minkov I, Hille J. Hydrogen peroxide-induced cell death in Arabidopsis: transcriptional and mutant analysis reveals a role of an oxoglutarate-dependent dioxygenase gene in the cell death process. IUBMB life. 2005;57(3):181–8. doi: 10.1080/15216540500090793 16036580

[75] Li W, Han Y, Tao F, Chong K. Knockdown of SAMS genes encoding S-adenosyl-l-methionine synthetases causes methylation alterations of DNAs and histones and leads to late flowering in rice. Journal of plant physiology. 2011;168(15):1837–43. doi: 10.1016/j.jplph.2011.05.020 21757254

[76] Hong Y, Zhang Y, Sinumporn S, Yu N, Zhan X, Shen X, et al. Premature leaf senescence 3, encoding a methyltransferase, is required for melatonin biosynthesis in rice. The Plant Journal. 2018. doi: 10.1111/tpj.13995 29901843

