# Correction: Mitochondrial phosphate transporter and methyltransferase genes contribute to Fusarium head blight Type II disease resistance and grain development in wheat

**DOI:** 10.1371/journal.pone.0308276

**Published:** 2024-07-30

**Authors:** Keshav B. Malla, Ganesh Thapa, Fiona M. Doohan

In the abstract section, there is an error in the fourth sentence. The correct sentence is: Virus-induced gene silencing (VIGS) of either TaMPT or TaSAM enhanced the susceptibility of cv. CM82036 to FHB disease, enhancing disease spread (Type II disease resistance).
