# Supplementary figures and images for: Mitochondrial phosphate transporter and methyltransferase genes contribute to Fusarium head blight Type II disease resistance and grain development in wheat
Source: PLoS One. 2021 Oct 14;16(10):e0258726. doi: 10.1371/journal.pone.0258726 (PMC8516198; doi:10.1371/journal.pone.0258726)

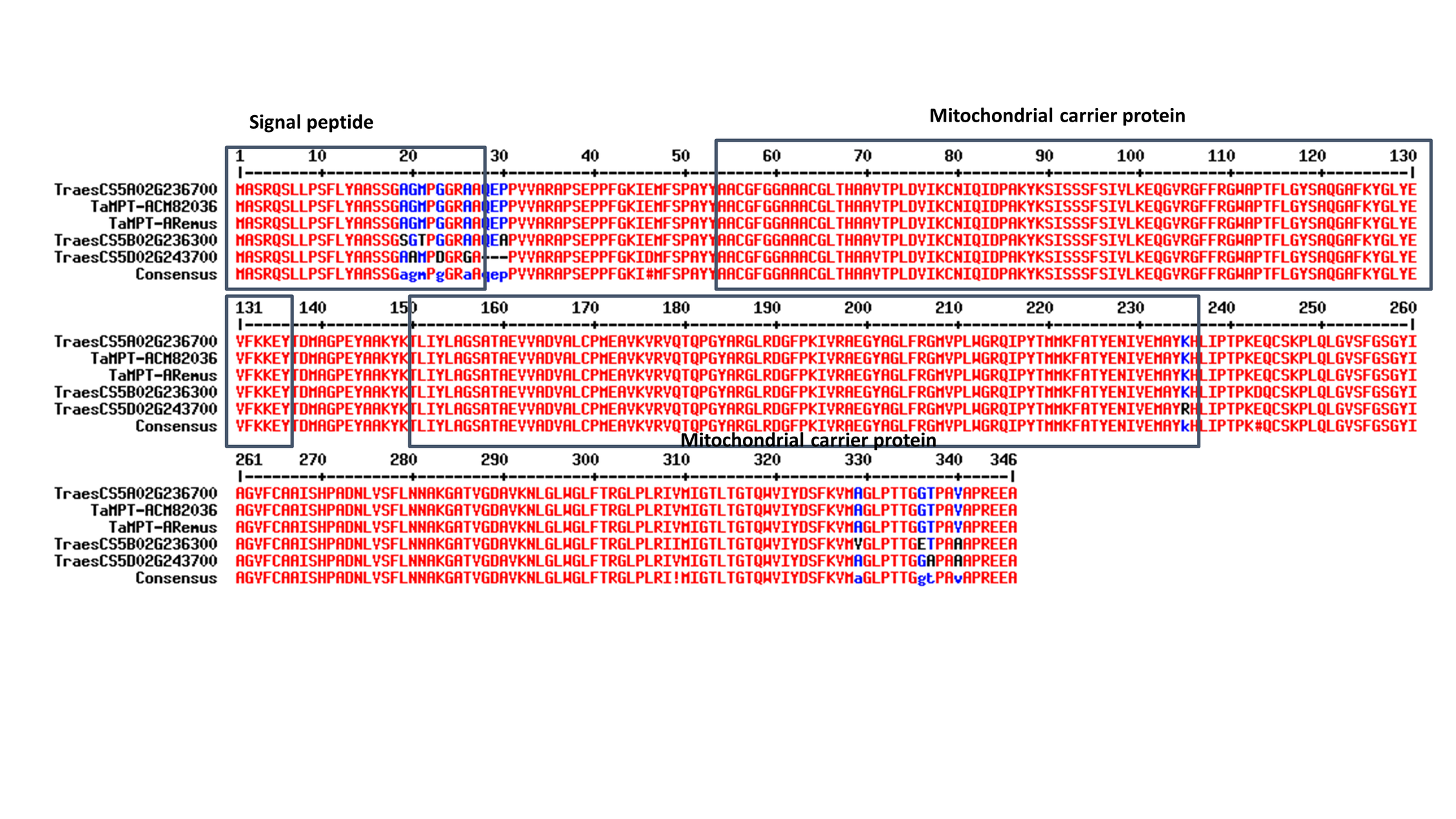

Supplement: S1 Fig — (TIF) [file pone.0258726.s001.tif]

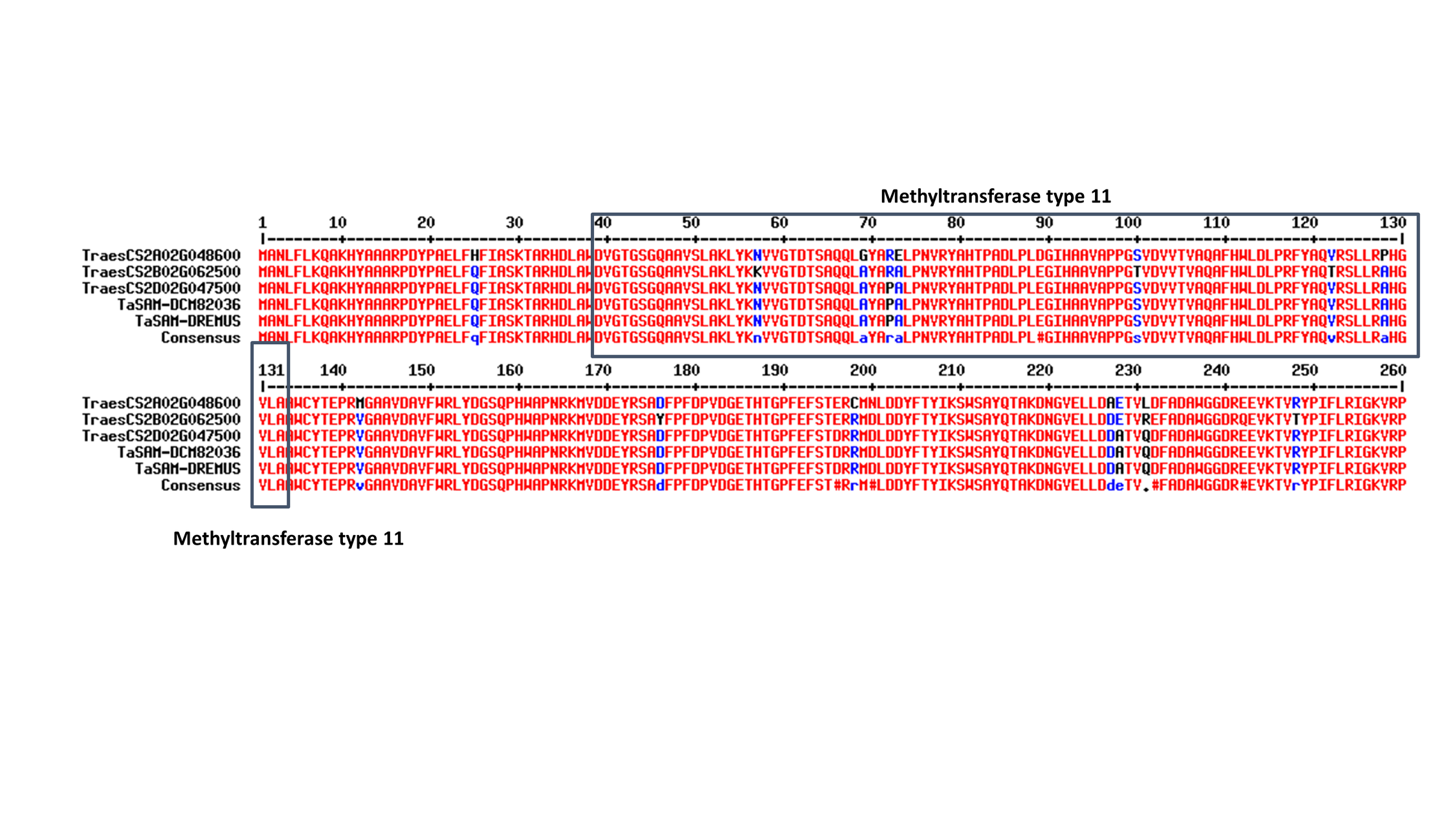

Supplement: S2 Fig — (TIF) [file pone.0258726.s002.tif]

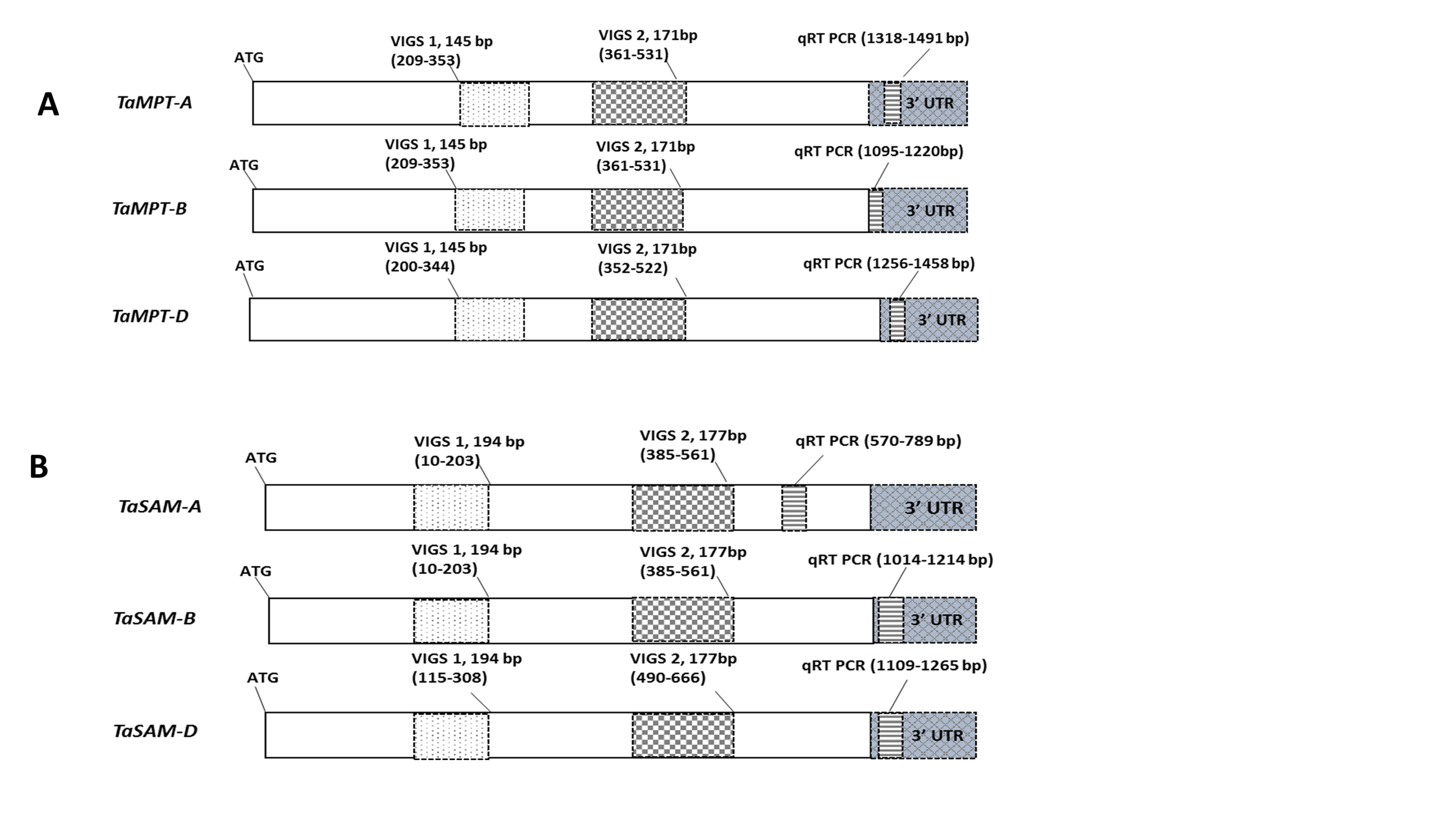

Supplement: S3 Fig — (A) TaMPT (chromosomes 5A, 5B and 5D). (B) TaSAM (chromosomes 2A, 2B and 2D). (TIF) [file pone.0258726.s003.tif]

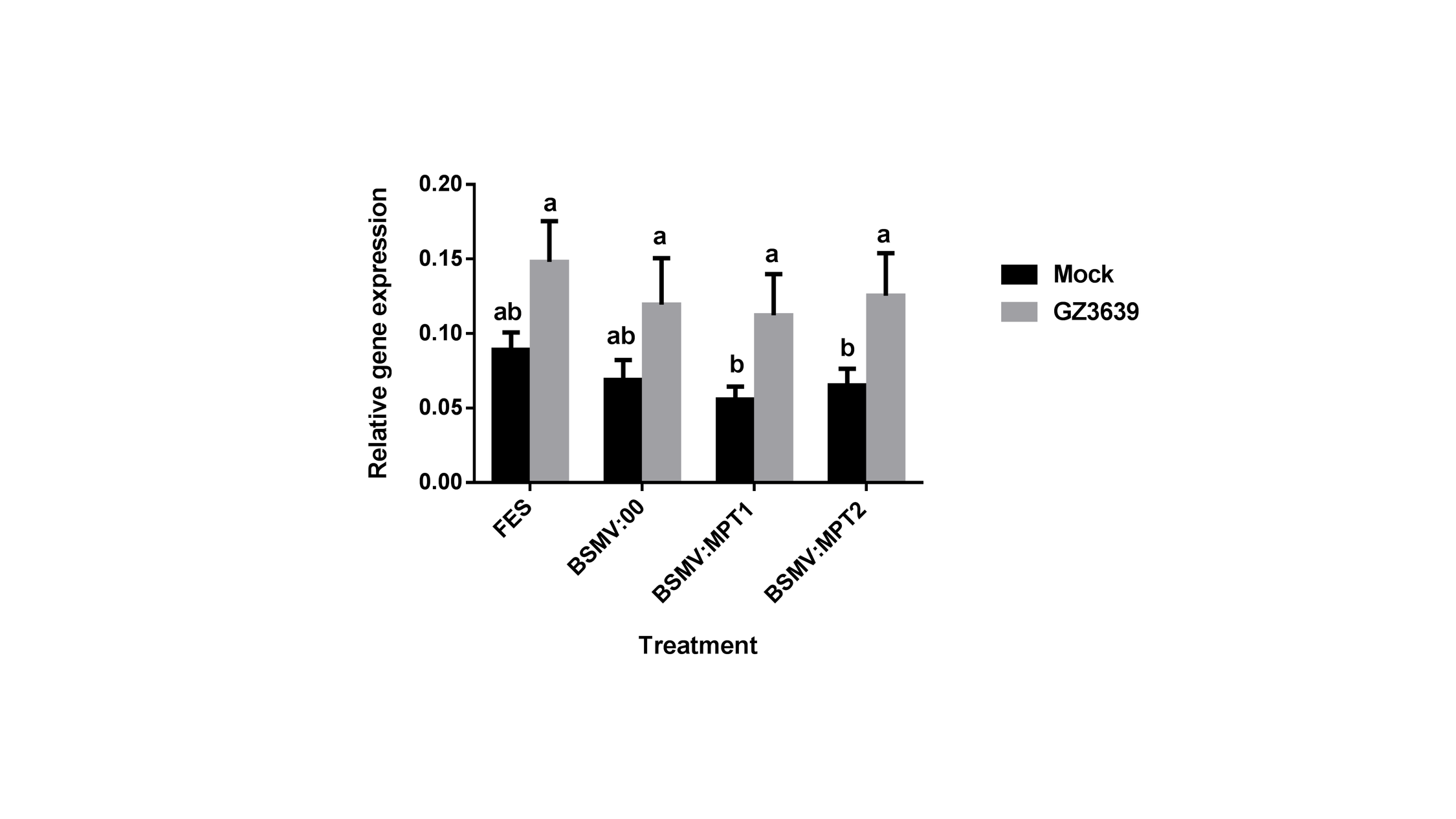

Supplement: S4 Fig — Flag leaves of wheat cv. CM82036 were rub-inoculated at growth stage 47 [36] just before the emergence of the first wheat head with representing either FES (VIGS buffer), in vitro transcribed RNAs BSMV:00 (empty vector) or BSMV: MPT1 or BSMV: MPT2 (construct targeting TaMPT). At mid-anthesis (growth stage 65) [36] two central spikelets of heads were inoculated with either conidia of F. graminearum strain GZ3639 or Tween-20 (mock treatment), as previously described [9]. After 24h, the third spikelet above the treated spikelets was harvested for gene expression analysis. The expression of TaMPT on chromosome 2 was quantified by real-time PCR analysis using wheat α-tubulin, YLS8 and TaPP2AA3 housekeeping genes (average of [2^-(CT target- CT α-tubulin)], [2^-(CT target- CT YLS8)] and [2^-(CT target- CT PP2AA3)] [48]. Gene expression data represents from the 60 heads per treatment combination (5 bulk RNA from four heads). Bars in graphs indicate standard error of the mean (SEM). Treatments with the same letter are not significantly different (P > 0.05). (TIF) [file pone.0258726.s004.tif]

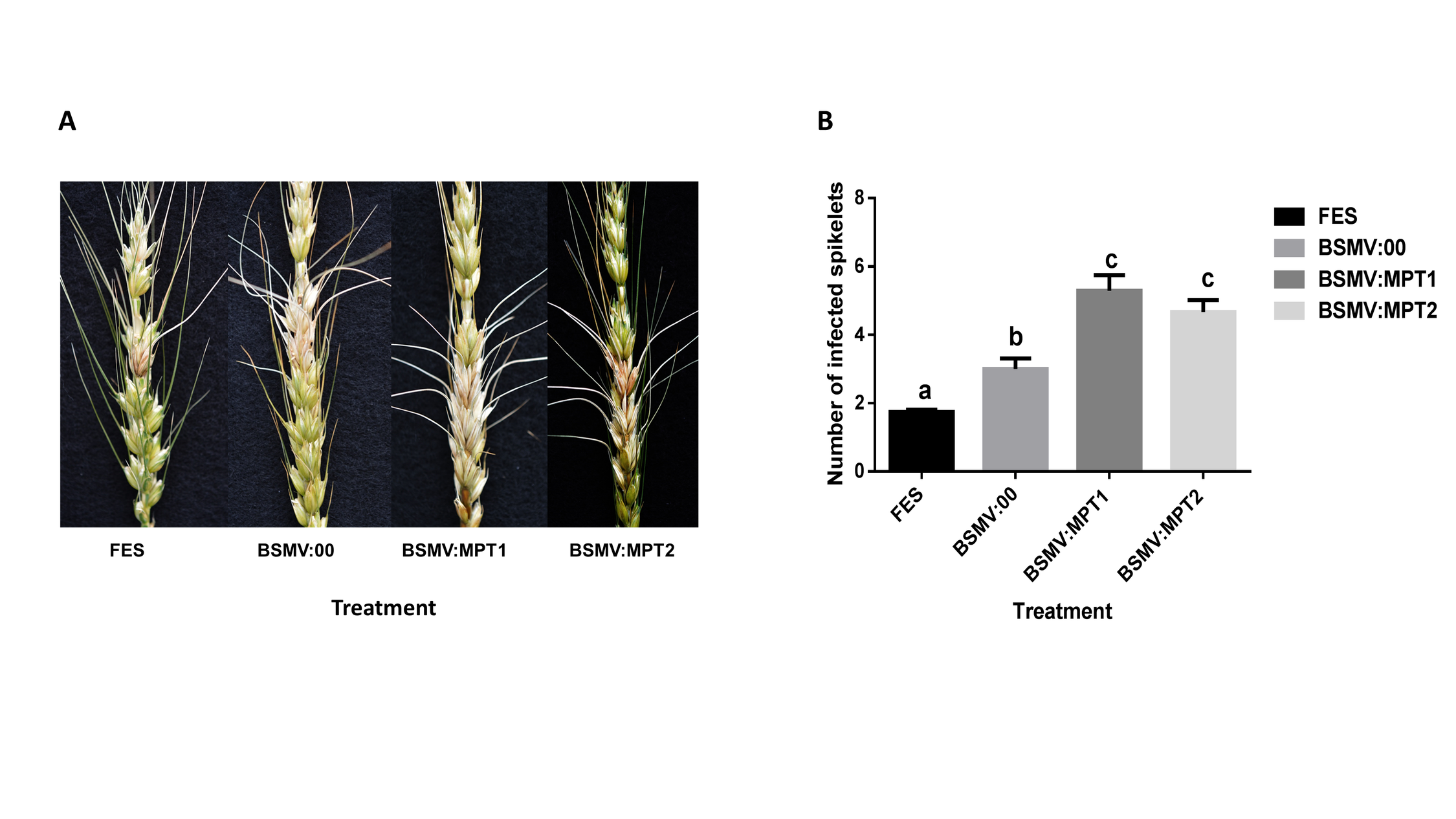

Supplement: S5 Fig — Flag leaves of wheat cv. CM82036 were rub-inoculated at growth stage 47 [36] just before the emergence of the first wheat head with representing either FES (VIGS buffer), in vitro transcribed RNAs BSMV:00 (empty vector) or BSMV: MPT1 or BSMV:MPT2 (construct targeting TaMPT). At mid-anthesis (growth stage 65) [36] two central spikelets of heads were inoculated with either conidia of F. graminearum strain GZ3639 or 0.02% Tween-20 (mock treatment), as previously described [9]. Disease symptoms were scored at 21 days post-treatment. (A) Images displaying typical disease symptoms at 21 days post-Fusarium treatment at silenced plants compared to mock (virus) treated samples. B) Quantification of the number of diseased spikelets per head in cv. CM82036 at 21 days post-treatment. Disease results represents mean data obtained from 60 heads (20 heads per treatment combination in each of three trials). Bars in graphs indicate standard error of the mean (SEM). Treatments with the same letter are not significantly different (P > 0.05). (TIF) [file pone.0258726.s005.tif]

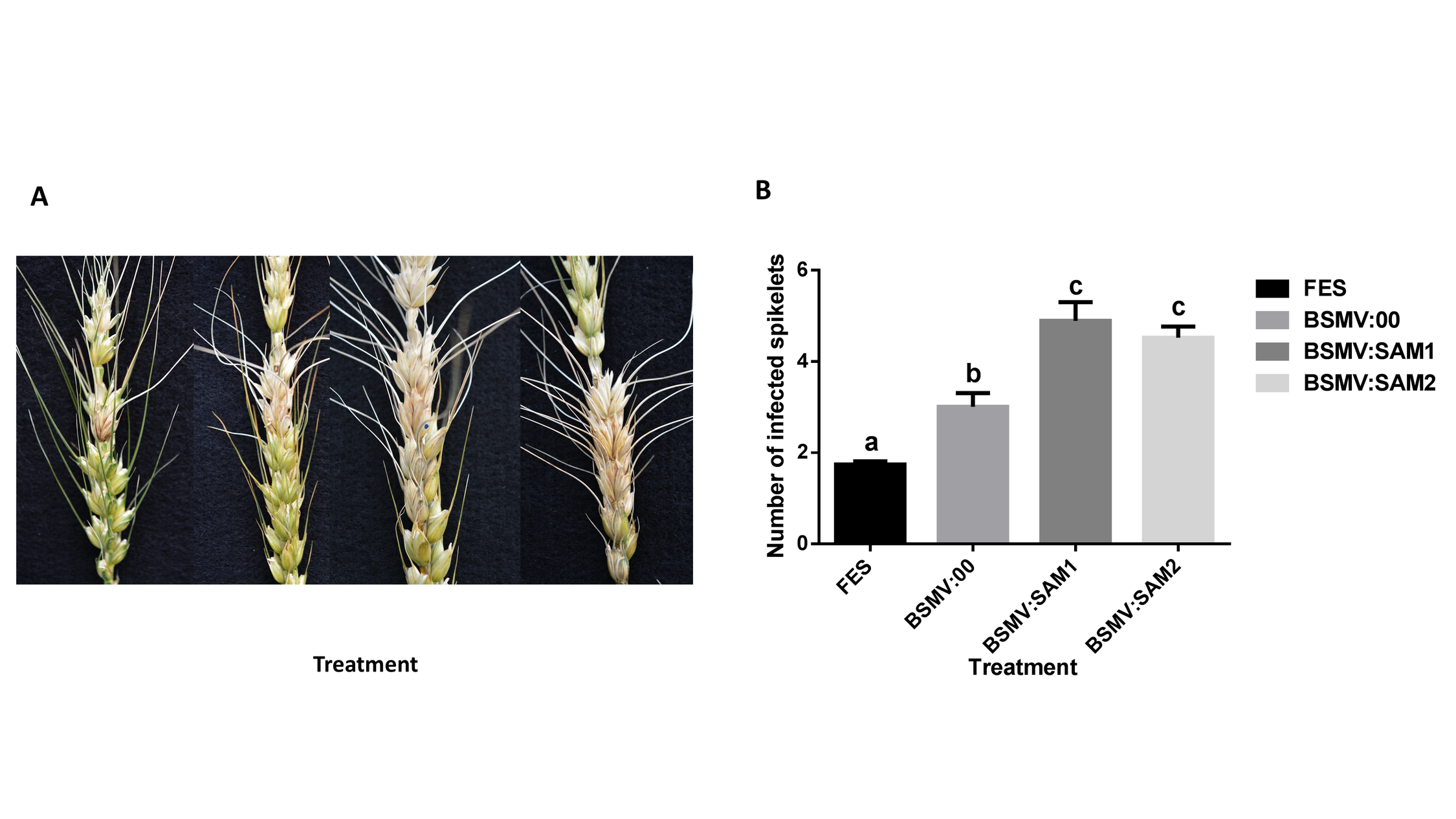

Supplement: S6 Fig — Flag leaves of wheat cv. CM82036 were rub-inoculated at growth stage 47 [36] just before the emergence of the first wheat head with representing either FES (VIGS buffer), in vitro transcribed RNAs BSMV:00 (empty vector) or BSMV: SAM1 or BSMV:SAM2 (construct targeting TaSAM). At mid-anthesis (growth stage 65) [36] two central spikelets of heads were inoculated with either conidia of F. graminearum strain GZ3639 or 0.02% Tween-20 (mock treatment), as previously described [9]. Disease symptoms were scored at 21 days post-treatment. (A) Images displaying typical disease symptoms at 21 days post-Fusarium treatment at silenced plants compared to mock (virus) treated samples. (B) Quantification of the number of diseased spikelets per head in cv. CM82036 at 21 days post-treatment. Disease results represents mean data obtained from 60 heads (20 heads per treatment combination in each of three trials). Bars in graphs indicate standard error of the mean (SEM). Treatments with the same letter are not significantly different (P > 0.05). (TIF) [file pone.0258726.s006.tif]
