# Supplementary material for: Mitochondrial phosphate transporter and methyltransferase genes contribute to Fusarium head blight Type II disease resistance and grain development in wheat
Source: PLoS One. 2021 Oct 14;16(10):e0258726. doi: 10.1371/journal.pone.0258726 (PMC8516198; doi:10.1371/journal.pone.0258726)
Supplement: S1 Table — (DOCX) [file pone.0258726.s007.docx]

**Table S1.** Experimental design for plant experiments.

| **Experiment** | **No of independent trials^a^** | **No of wheat cultivars** | **No of plants/heads per treatment combination per cultivar^b, c^** | **No of samples used for disease and grain analysis per cultivar** | **RNA samples per treatment combination^b^** |
| --- | --- | --- | --- | --- | --- |
| Adult plant DON and FHB time course experiment | 3 | 2 (Remus and CM82036) | 4/8 per trial; total of 12/24 for the experiment | --- | 1 sample per trial (pooled from RNA extracted from the 8 heads); total = 3 across all 3 trials |
| Virus-induced gene silencing experiment | 3 | 1 (CM82036) | 10/20 per trial: total of 30/60 for the experiment | 10/20 per trial: total of 30/60 for the experiment | 5 samples per trial (each pooled from RNA extracted from 1 spikelet per each of 4 treated heads within the trial); total = 15 across all 3 trials |

**^a^**Note that each replicate trial within an experiment was conducted at a different time and with independently produced fungal inoculum where relevant.

**^b^**Treatments for the adult plant DON and FHB time course experiment: Negative control (Tween20), DON, *Fusarium graminearum.*

**^c^**Treatment combinations for the VIGS experiment: BSMV:00 + Tween20, BSMV:PDS + Tween20, BSMV:SAM1 + Tween20, BSMV:SAM2 + Tween20, BSMV:MPT1 + Tween20, BSMV:MPT2 + Tween20, BSMV:00 + *F. graminearum*, BSMV:PDS + + *F. graminearum*, BSMV:SAM1 + *F. graminearum*, BSMV:SAM2 + + *F. graminearum*, BSMV:MPT1 + *F. graminearum*, BSMV:MPT2 + *F. graminearum.*
