# Supplementary material for: Mitochondrial phosphate transporter and methyltransferase genes contribute to Fusarium head blight Type II disease resistance and grain development in wheat
Source: PLoS One. 2021 Oct 14;16(10):e0258726. doi: 10.1371/journal.pone.0258726 (PMC8516198; doi:10.1371/journal.pone.0258726)
Supplement: S2 Table — (DOCX) [file pone.0258726.s008.docx]

**Table S2.** List of primers used in this study.

| **Gene** | **Gene/variant ID** | **Primer name and sequence (5’-3’)** | **Application** |  |
| --- | --- | --- | --- | --- |
| *TaSAM-A* | TraesCS2A02G048600 | 2AF5:GAGTTCTCCACGGAGCGAT  2AR5:TCTGATTCTTAAGGCCTCACC | Homoeolog-specific gene expression primers |  |
| *TaSAM-B* | TraesCS2B02G062500 | 2BF2:TACTACCATCCCCTCCTATTCA  2BR2:AACTGTCAGAAAAATCGACTGG |  |  |
| *TaSAM-D* | TraesCS2D02G047500 | 2DF4: CTATCCCGTTCCTATTCCTATTC  2DR4:TCAGCAACACCGCAAAAATAAT |  |  |
| *TaMPT*-A | TraesCS5A02G236700 | 5AF2:GATGTTTGTAACAGTTTTCCTCG  5AR2: GGGACGAAAGACTGTTTTGAT |  |  |
| *TaMPT*-B | TraesCS5B02G236300 | 5BF3: TTGGACTGCCAACAACCGGTGA  5BR3: ACTACAGATTATGCGATGTCG |  |  |
| *TaMPT*-D | TraesCS5D02G243700 | 5DF2:TCGCATGATCTGTAATAGATTGA  5DR2:AGGCGAAAGACTGTTTTGAGGA |  |  |
| *TaSAM-D* | TraesCS2D02G047500 | 2DF1: CTGTGGTCCCAGTTCAAGC  2DR3: GGAATAGGAATAGGAACGGGATA | 2D specific primers  for *TaSAM-D* gene and promoter cloning |  |
| *TaMPT-A* | TraesCS5A02G236700 | 5AF2: CTGATCGTGACACTTTCAGTA  5AR2: TTTGGAACCTTAATGACCAAT | 5A specific primers  for *TaMPT-A* gene cloning |  |
| *TaMPT-A* | TraesCS5A02G236700 | 5AF4: GCTAAGGGTGTCATTGGTTGA  5AR2: AGTACGCCGGCGAGAACATCTCG | 5A specific primers  for *TaMPT-A* promoter cloning |  |
| *TaSAM* |  | 2ABDF1:CTGTTCCTGAAGCAGGCGAAGC  2ABDR1: CAGCTGCTGCGCGCTCGTGTC | VIGS fragment 1 targeting *TaSAM* (used to generate BSMV:SAM1) |  |
| *TaSAM* |  | 2ABDF2: CACGGCGTCCTCGCCGCATG  2ABDR2: GTGGGTCTCGCCGTCGAC | VIGS fragment 2 targeting *TaSAM* (used to generate BSMV:SAM2) |  |
| *TaMPT* |  | 5ABDF1: CAGGGCGCGTTCAAGTACGG  5ABDR1:CCGCACCTTGACGGCCTCCATG | VIGS fragment 1 targeting *TaMPT* (used to generate BSMV:MPT1) |  |
| *TaMPT* |  | 5ABDF2: CCGTGACGCCGCTCGATGTC  5ABDR2: TACCCCAGGAACGTGGGC | VIGS fragment 2 targeting *TaMPT* (used to generate BSMV:MPT2) |  |
| pGamma |  | pGamma F:TGATGATTCTTCTTCCGTTGC  pGammaR:TGGTTTCCAATTCAGGCATCG | Sequencing of VIGS constructs within BSMV |  |
| *TaMPT* | TraesCS2A02G316900  TraesCS2B02G335500  TraesCS2D02G314800 | 2ABDF1: CTGCGTATCGTGATGATTG  2ABDR1: TAGTTGGCAGTCCAACCATGA | *TaMPT* Primers used to check off-target effects of *TaMPT* VIGS |  |
