# Supplementary material for: Mitochondrial phosphate transporter and methyltransferase genes contribute to Fusarium head blight Type II disease resistance and grain development in wheat
Source: PLoS One. 2021 Oct 14;16(10):e0258726. doi: 10.1371/journal.pone.0258726 (PMC8516198; doi:10.1371/journal.pone.0258726)
Supplement: S3 Table — (DOCX) [file pone.0258726.s009.docx]

**Table S3.** DNA sequence similarity between the conserved domains of *TaMPT-A* from wheat cv. CM82036 and homoeologs from cvs. Remus and Chinese spring.

| **Wheat cultivar** |  |  | **Gene** | **Identity to *TaMPT-5A* from cv. CM82036 (%)** | **Query coverage (bp)** |
| --- | --- | --- | --- | --- | --- |
| CM82036 |  |  | *TaMPT-A* | 100 | 1-1041 |
| Remus |  |  | *TaMPT-A* | 100 | 1-1041 |
| Chinese Spring |  |  | *TaMPT-A* | 100 | 1-1041 |
| Chinese Spring |  |  | *TaMPT-B* | 98.07 | 1-1041 |
| Chinese Spring |  |  | *TaMPT-D* | 98.17 | 1-1041 |
