# Supplementary material for: Mitochondrial phosphate transporter and methyltransferase genes contribute to Fusarium head blight Type II disease resistance and grain development in wheat
Source: PLoS One. 2021 Oct 14;16(10):e0258726. doi: 10.1371/journal.pone.0258726 (PMC8516198; doi:10.1371/journal.pone.0258726)
Supplement: S4 Table — (DOCX) [file pone.0258726.s010.docx]

**Table S4.** Protein sequence similarity of *TaMPT-A* from wheat cv. CM82036 with *TaMPT-A* Remus with 5A, 5B, and 5D homoeologs of Chinese spring.

| **Wheat cultivar** | **Gene** | **Percent Identity** | **Protein Length (amino acid)** |
| --- | --- | --- | --- |
| CM82036 | *TaMPT-A* | 100 | 346 |
| Remus | *TaMPT-A* | 100 | 346 |
| Chinese Spring | *TaMPT-A* | 100 | 346 |
| Chinese Spring | *TaMPT-B* | 98.26 | 346 |
| Chinese Spring | *TaMPT-D* | 98.26 | 343 |
