# Supplementary material for: Mitochondrial phosphate transporter and methyltransferase genes contribute to Fusarium head blight Type II disease resistance and grain development in wheat
Source: PLoS One. 2021 Oct 14;16(10):e0258726. doi: 10.1371/journal.pone.0258726 (PMC8516198; doi:10.1371/journal.pone.0258726)
Supplement: S5 Table — (DOCX) [file pone.0258726.s011.docx]

**Table S5**. Domain position and sub-cellular localisation of *TaMPT* gene and their homoeologs.

| **Gene** | **Gene ID** | **Protein Length (aa)** | **Domain/position** | | | **Superfamily** | **Subcellular localisation** |
| --- | --- | --- | --- | --- | --- | --- | --- |
| *TaMPT-A* | TraesCS5A02G236700 | 346 | Signal peptide  (1-27) | Mitochondrial carrier protein  ([IPR018108](http://www.ebi.ac.uk/interpro/entry/IPR018108))  (54-137) | Mitochondrial carrier protein  ([IPR018108](http://www.ebi.ac.uk/interpro/entry/IPR018108))  (150-236) | Mitochondrial family  ([IPR023395](http://www.ebi.ac.uk/interpro/entry/IPR023395))  (52-323) | Mitochondrial |
| *TaMPT-B* | TraesCS5B02G236300 | 346 | Signal peptide  (1-26) | Mitochondrial carrier protein  ([IPR018108](http://www.ebi.ac.uk/interpro/entry/IPR018108))  (54-137) | Mitochondrial carrier protein  ([IPR018108](http://www.ebi.ac.uk/interpro/entry/IPR018108))  (150-236) | Mitochondrial family  ([IPR023395](http://www.ebi.ac.uk/interpro/entry/IPR023395))  (52-323) | Mitochondrial |
| *TaMPT-D* | TraesCS5D02G243700 | 343 | Signal peptide  (1-20) | Mitochondrial carrier protein  ([IPR018108](http://www.ebi.ac.uk/interpro/entry/IPR018108))  (51-134) | Mitochondrial carrier protein  ([IPR018108](http://www.ebi.ac.uk/interpro/entry/IPR018108))  (147-232) | Mitochondrial family  ([IPR023395](http://www.ebi.ac.uk/interpro/entry/IPR023395))  (49-320) | Mitochondrial |
