# Supplementary material for: Mitochondrial phosphate transporter and methyltransferase genes contribute to Fusarium head blight Type II disease resistance and grain development in wheat
Source: PLoS One. 2021 Oct 14;16(10):e0258726. doi: 10.1371/journal.pone.0258726 (PMC8516198; doi:10.1371/journal.pone.0258726)
Supplement: S6 Table — (DOCX) [file pone.0258726.s012.docx]

**Table S6.** DNA sequence similarity between the conserved domains of *TaSAM-D* from wheat cv. CM82036 and homoeologs from cvs. Remus and Chinese spring

| **Wheat cultivar** |  |  |  | **Gene** | **Identity to *TaSAM-2D* from cv. CM82036 (%)** | **Query coverage (bp)** |
| --- | --- | --- | --- | --- | --- | --- |
| CM82036 |  |  |  | *TaSAM-D* | 100 | 1-783 |
| Remus |  |  |  | *TaSAM-D* | 100 | 1-783 |
| Chinese spring |  |  |  | *TaSAM-A* | 95.91 | 1-783 |
| Chinese spring |  |  |  | *TaSAM-B* | 96.43 | 1-783 |
| Chinese spring |  |  |  | *TaSAM-D* | 100 | 1-783 |
|  |  |  |  |  |  |  |
