# Supplementary material for: Mitochondrial phosphate transporter and methyltransferase genes contribute to Fusarium head blight Type II disease resistance and grain development in wheat
Source: PLoS One. 2021 Oct 14;16(10):e0258726. doi: 10.1371/journal.pone.0258726 (PMC8516198; doi:10.1371/journal.pone.0258726)
Supplement: S7 Table — (DOCX) [file pone.0258726.s013.docx]

**Table S7.** Protein sequence similarity of *TaSAM-D* from wheat cv. CM82036 with *TaSAM-D* Remus, 2A, 2B, and 2D homoeologs of Chinese spring

| **Wheat Cultivar** | **Gene** | **Percent Identity** | **Protein Length**  **(amino acid)** |
| --- | --- | --- | --- |
| CM82036 | *TaSAM-D* | 100 | 260 |
| Remus | *TaSAM-D* | 100 | 260 |
| Chinese spring | *TaSAM-A* | 96.54 | 260 |
| Chinese spring | *TaSAM-B* | 96.93 | 260 |
| Chinese spring | *TaSAM-D* | 100 | 260 |
