# Supplementary material for: Mitochondrial phosphate transporter and methyltransferase genes contribute to Fusarium head blight Type II disease resistance and grain development in wheat
Source: PLoS One. 2021 Oct 14;16(10):e0258726. doi: 10.1371/journal.pone.0258726 (PMC8516198; doi:10.1371/journal.pone.0258726)
Supplement: S8 Table — (DOCX) [file pone.0258726.s014.docx]

**Table S8.** Domain position and sub-cellular localisation of *TaSAM* gene and their homoeologs

| **Gene** | **Gene ID** | **Protein length (aa)** | **Domain/position** | **superfamily** | **Subcellular localisation** |
| --- | --- | --- | --- | --- | --- |
| *TaSAM-A* | TraesCS2A02G048600 | 260 | Methyltransferase type 11  (IPR013216)    (39-133) | S-adenosyl-L-methionine-dependent methyltransferase  ( IPR029063)  (4-182) | Cytoplasmic |
| *TaSAM-B* | TraesCS2B02G062500 | 260 | Methyltransferase type 11  (IPR013216)    (39-133) | S-adenosyl-L-methionine-dependent methyltransferase  ( IPR029063)  (4-190) | Cytoplasmic |
| *TaSAM-D* | TraesCS2D02G047500 | 260 | Methyltransferase type 11  (IPR013216)    (39-133) | S-adenosyl-L-methionine-dependent methyltransferase  ( IPR029063)  (19-202) | Cytoplasmic |

|  |
| --- |
