# Supplementary material for: Mitochondrial phosphate transporter and methyltransferase genes contribute to Fusarium head blight Type II disease resistance and grain development in wheat
Source: PLoS One. 2021 Oct 14;16(10):e0258726. doi: 10.1371/journal.pone.0258726 (PMC8516198; doi:10.1371/journal.pone.0258726)
Supplement: S9 Table — (DOCX) [file pone.0258726.s015.docx]

**Table S9.** Specificity of the mitochondrial phosphate transporter (*TaMPT*) and methyltransferase (*TaSAM*) construct used for VIGS.

| Homoeologous locus | Construct BSMV:MPT1 (171bp)  Homology %*^a^*  (Silencing*^b^*) | | Construct BSMV:MPT2 (145bp)  Homology %*^a^*  (Silencing*^b^*) | | Homoeologous locus | Construct BSMV:SAM1 (194bp)  Homology %*^c^*  (Silencing*^d^*) | Construct BSMV:SAM2 (177bp)  Homology %*^c^*  (Silencing*^d^*) |
| --- | --- | --- | --- | --- | --- | --- | --- |
| 5A | 100 (yes) |  | 100  (Yes) |  | 2A | 100  (Yes) | 97.2  (Yes) |
| 5B | 100  (Yes) |  | 100  (Yes) |  | 2B | 97.2  (Yes) | 96.6  (Yes) |
| 5D | 100  (Yes) |  | 100  (Yes) |  | 2D | 97.9  (Yes) | 100  (Yes) |

*^a^*Homology of *TaMPT* construct to the cv. Chinese Spring genome sequence.

*^b^*Based on qRT-PCR analysis, using primers designed based on *TaMPT* and wheat cv. Chinese spring genome sequences.

*^c^*Homology of *TaSAM* construct to the cv. Chinese Spring genome sequence.

*^d^*Based on qRT-PCR analysis, using primers designed based on *TaSAM* and wheat cv. Chinese spring genome sequences.
